# Comparison of Cu^+^, Ag^+^, and Au^+^ Ions as Ionization Agents of Volatile Organic Compounds at Subatmospheric Pressure

**DOI:** 10.1021/jasms.3c00370

**Published:** 2024-01-24

**Authors:** Monika Koktavá, Vadym Prysiazhnyi, Jan Preisler, Antonín Bednařík

**Affiliations:** Department of Chemistry, Faculty of Science, Masaryk University, 625 00 Brno, Czech Republic

## Abstract

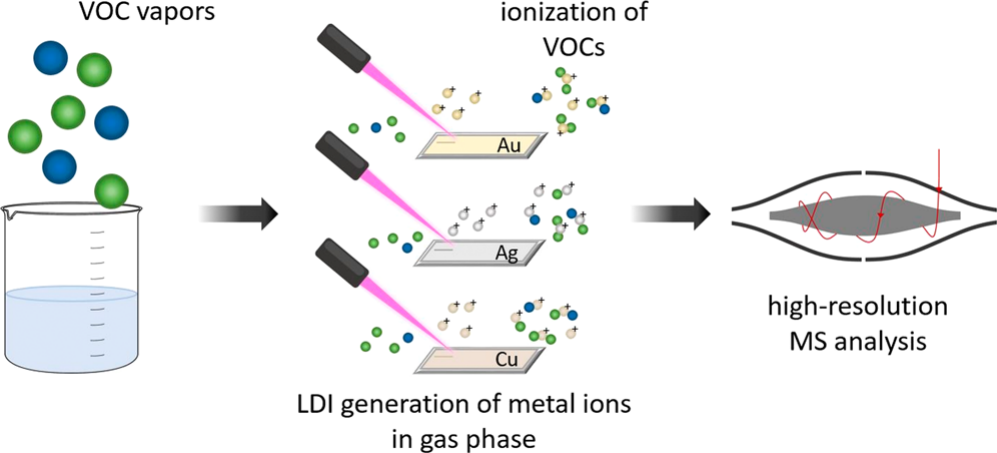

Ionization of volatile organic compounds (VOCs) by coinage metal ions (Cu^+^, Ag^+^, and Au^+^) generated by laser desorption and ionization (LDI) of a metal nanolayer in subatmospheric conditions is explored. The study was performed in a commercial subatmospheric dual MALDI/ESI ion source. Five compounds representing different VOC classes were chosen for a detailed study of the metal ionization mechanism: ethanol, acetone, acetic acid, xylene, and cyclohexane. In the gas phase, ion molecular complexes of all three metal ions were formed, typically with two ligand molecules. The successful detection of the metal complexes with VOCs strongly depended on the applied voltages across the ion source, minimizing the in-source fragmentation. The employed orbital trap with ultrahigh resolving power and sub-parts-per-million mass accuracy allowed unambiguous identification of the formed complexes based on their molecular formulas. The detection limits of the studied compounds in the gas were in the range 0.1–1.4 nmol/L. Compared to Cu^+^ and Ag^+^ ions, Au^+^ ions exhibited the highest reactivity, often complicating spectra by side products of reactions. On the other hand, they also allowed detecting saturated hydrocarbons, which did not produce any signals with Ag^+^ and Cu^+^.

## Introduction

Volatile organic compounds (VOCs) encompass many different classes of organic substances characterized by a high vapor pressure at room temperature. Some of them are naturally occurring in the environment, but a large quantity of VOCs is produced and used by humans. VOCs produced by industry, extensive use of solvents, and fuel combustion contribute to outdoor air pollution. Common sources of VOCs in indoor environments include cleaning agents, cosmetics, paints, printers, adhesives, and furniture. Prolonged exposure to these substances may have adverse effects on human health, ranging from irritation and cognitive impairment to neuro- or genotoxicity and carcinogenicity.^1^ Therefore, the importance of VOC monitoring in both indoor and outdoor air is at hand.

In addition, VOC analysis can also serve diagnostic purposes, aiding in the detection of various diseases or metabolism changes. A typical example is breath analysis, a noninvasive method that can provide valuable information about the health status of patients.^2^ The connection between breath composition and diseases such as diabetes, lung cancer, cystic fibrosis, asthma, and liver diseases has been studied.^3−7^ Additionally, microorganisms can be monitored based on the VOC profiles they produce.^2,8,9^

Mass spectrometry (MS) techniques for the analysis of VOCs offer a rapid and sensitive determination in real time with minimal sample preparation requirements. Selected ion flow tube (SIFT) MS uses a discharge in an ion source to generate a mixture of positive and negative ions. Reaction ions, typically H_3_O^+^, NO^+^, or O_2_^+•^, are selected by a quadrupole filter due to their minimal reactivity with the main components of air. Analyte molecules carried into the flow tube react with the selected reaction ions, giving rise to characteristic products detected along with unused reaction ions.^10,11^ Reactions occurring in SIFT MS include proton transfer, hydride ion transfer, charge transfer, and association. These reactions may occur simultaneously, complicating spectra and interpretation of the results. A technique similar to SIFT is proton transfer reaction (PTR) MS utilizing ion–molecule reactions of VOCs with H_3_O^+^ or NH_4_^+^ ions, resulting in the generation of primary protonated products [M + H]^+^. Another ambient technique suitable for the analysis of gaseous samples is secondary electrospray ionization (SESI) MS. The classical ESI source is modified to include a second capillary for the introduction of the sample. ESI generated ions then undergo ion–molecule reactions with analytes leading to the formation of protonated [M + H]^+^ or deprotonated [M – H]^−^ ions in positive and negative modes, respectively.^12,13^ A promising technique for the analysis of VOCs utilizing dielectric barrier discharge ionization has been commercialized recently as soft ionization by chemical reaction in transfer (SICRIT).^14^ The sample is introduced through a quartz capillary to a cold plasma generated between two concentric electrodes. The short duration of sample exposure to plasma results in ionization with minimal fragmentation and formation of predominantly [M + H]^+^ ions.^15,16^ Detection limits for these methods range from parts-per-billion to parts-per-trillion, depending on the experimental conditions and analytes.^14,17,18^

Recently, we have introduced an MS technique for VOC analysis based on ionization of VOCs by Au^+^ ions generated by subatmospheric laser desorption/ionization (subAP LDI).^19^ In the gaseous state, gold exhibits a unique set of chemical properties that are distinct from those in the bulk state. It is mainly due to the high reactivity and associated relativistic effects of Au^+^ ions.^20,21^ One of the interesting properties of the Au^+^ ion is the ability to form charged molecular–ion complexes, [Au + VOC]^+^, [Au + VOC + H_2_O]^+^, and [Au + 2VOC]^+^, with multiple classes of VOCs in subAP pressure, serving as a charging agent. However, this phenomenon is not limited to Au^+^ ions. The organometallic chemistry in the gas phase was extensively studied already in the 1980s. For example, reactions of Cu^+^ produced by laser ablation with various organic compounds were studied by ion cyclotron resonance (ICR) spectrometry.^22^ Reactions at low pressures (≲10^–7^ Torr) led to fragmentation of the examined compounds, while at higher pressures bonding of one or more molecules to the metal ion was observed, providing molecular weight information. Later, Li^+^ ions produced inside the thermionic source were used for ionizing organic compounds by forming simple ion–molecular complexes in the gas phase.^23,24^ Cu^+^ ions produced by laser ablation in a laser plasma ion source were used for the detection of octane and trinitrotoluene in the gas phase, and the reaction time of the complex formation between Cu^+^ ion and organic molecules was estimated to be about 1 ms at a pressure of 0.3 Torr.^25^ In both these examples, complexes containing molecules of water, [M + H_2_O]^+^, [M + 2H_2_O]^+^, and [M + VOC + H_2_O]^+^, were also present in the spectra, which was similar to our previous work with Au^+^ ions. In another work, an inductively coupled plasma (ICP) ion source coupled to the SIFT mass spectrometer was used to generate 20 positive ions of the main group elements, and their bonding to benzene was studied.^26^ Again, mostly complexes with one and two benzene ligands were observed; only Ca^+^, Sr^+^, and Ba^+^ exhibited an exceptional behavior and formed complexes with three benzene molecules. Several works focused on determining the role of relativistic effects in the reactivity of coinage metals going down the periodic table.^27−29^ Generally, these effects increase the reaction rates and binding energies of ligands with the third row transition metal cations in the gas phase.

In the current work, we further explore the technique of metal ionization in gas phase (MIG) inside the dual subAP matrix-assisted laser desorption/ionization/electrospray ionization (MALDI/ESI) ion source and compare the performance of three coinage metals: gold, silver and copper. The analytical utility is compared for VOCs belonging to different classes: alcohols, ketones, carboxylic acids, and saturated and aromatic hydrocarbons. Further, we estimate the limits of detection (LODs) and linear dynamic range of the technique and investigate the mechanism of ion–molecular reactions occurring in the ion source.

## Materials and Methods

### Chemicals

Ethanol, xylene, and acetic acid were purchased from Penta chemicals (Praha, Czech Republic); 1-propanol, 2-propanol, 1-pentanol, acetone, 2-butanone, 2-pentanone, 2-heptanone, propanoic acid, and pentanoic acid were purchased from Sigma-Aldrich (St. Louis, MO, USA); cyclohexane was from Lach-Ner, Ltd. (Neratovice, Czech Republic). 1-Butanol was purchased from Mach chemikalie spol. s r.o. (Slezska Ostrava, Czech Republic).

### Preparation of Metal Nanolayers

For the preparation of thin metal films, magnetron sputtering was used. The films were prepared on standard microscope slides cleaned with soapy water, deionized water, and ethanol. Film thicknesses of 6, 10, and 8 nm were chosen for Au, Ag, and Cu, respectively, as they were found to produce the highest M^+^ ion signals in LDI MS. Gold films were prepared using a commercial coater (Q150T ES Plus); silver and copper films were prepared in a laboratory-built vacuum chamber equipped with a 3 in. TORUS magnetron gun (Kurt J. Lesker, USA). A detailed description of the magnetron chamber, sputtering conditions, and optimization of film thickness is given in the Supporting Information.

### Mass Spectrometry

The measurements were performed with an ultrahigh resolution Q Exactive Plus mass spectrometer (Thermo Scientific, Germany) with a subAP MALDI/ESI ion source (MassTech, USA) equipped with a pulsed Nd:YAG laser (355 nm) operated at a repetition rate of 1 kHz. The metal-coated glass slide was mounted in a metal adapter and scanned with the laser in continuous raster mode. The target moved at a constant speed of 3.445 mm/min under the stationary laser beam. The width of the ablated lines, as observed by an optical microscope, ranged from 6 to 10 μm. The laser energy used for LDI of gold, silver, and copper layers was 0.24, 0.22, and 0.32 μJ/pulse, respectively. These energies were well above the threshold energy for metal desorption, allowing generation of a sufficient amount of metal ions in the gas phase.

**Figure 1:**
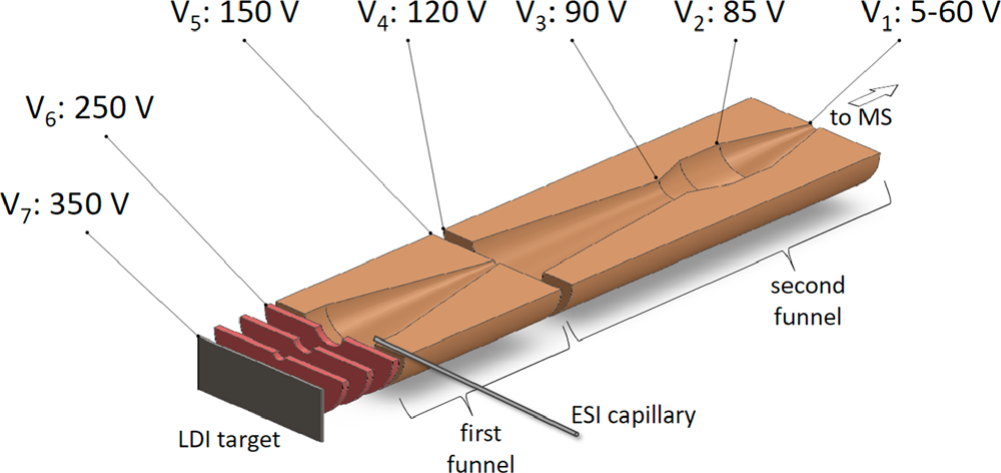
Schematics of the ion funnels with applied voltages.

The analyte was introduced into the ion source through a heated ESI capillary maintained at 300 °C to prevent the sorption of compounds on its inner surface. Samples were introduced in two ways: direct infusion of solution to the ESI capillary using a syringe equipped with a quartz capillary (i.d. 40 μm, o.d. 110 μm) or simply by placing an open beaker containing the VOC near the cone with the ESI capillary. The mass spectra were recorded in the *m*/*z* range from 50 to 750 Da with 140 000 resolution (at *m*/*z* 200 Da). During the measurements, the injection time (IT) for ion accumulation in the C-trap was set to 500 ms, and automatic gain control (AGC) was turned off in the leveraged mode. The pressure in the ion source was ∼390 Pa (2.9 Torr) for direct injection and for simply placing a beaker near the ESI capillary. The measurements were conducted in positive ion mode, and ions were guided to the mass spectrometer through a two-stage ion funnel. The schematics of the funnel and applied voltages are provided in Figure 1. Voltages *V*_2_–*V*_7_ were kept constant, while the voltage at the exit of the funnel, *V*_1_, was changed from 5 to 60 V.

## Results and Discussion

### Formation of Ion–Molecular Complexes of H_2_O with Metals

**Figure 2:**
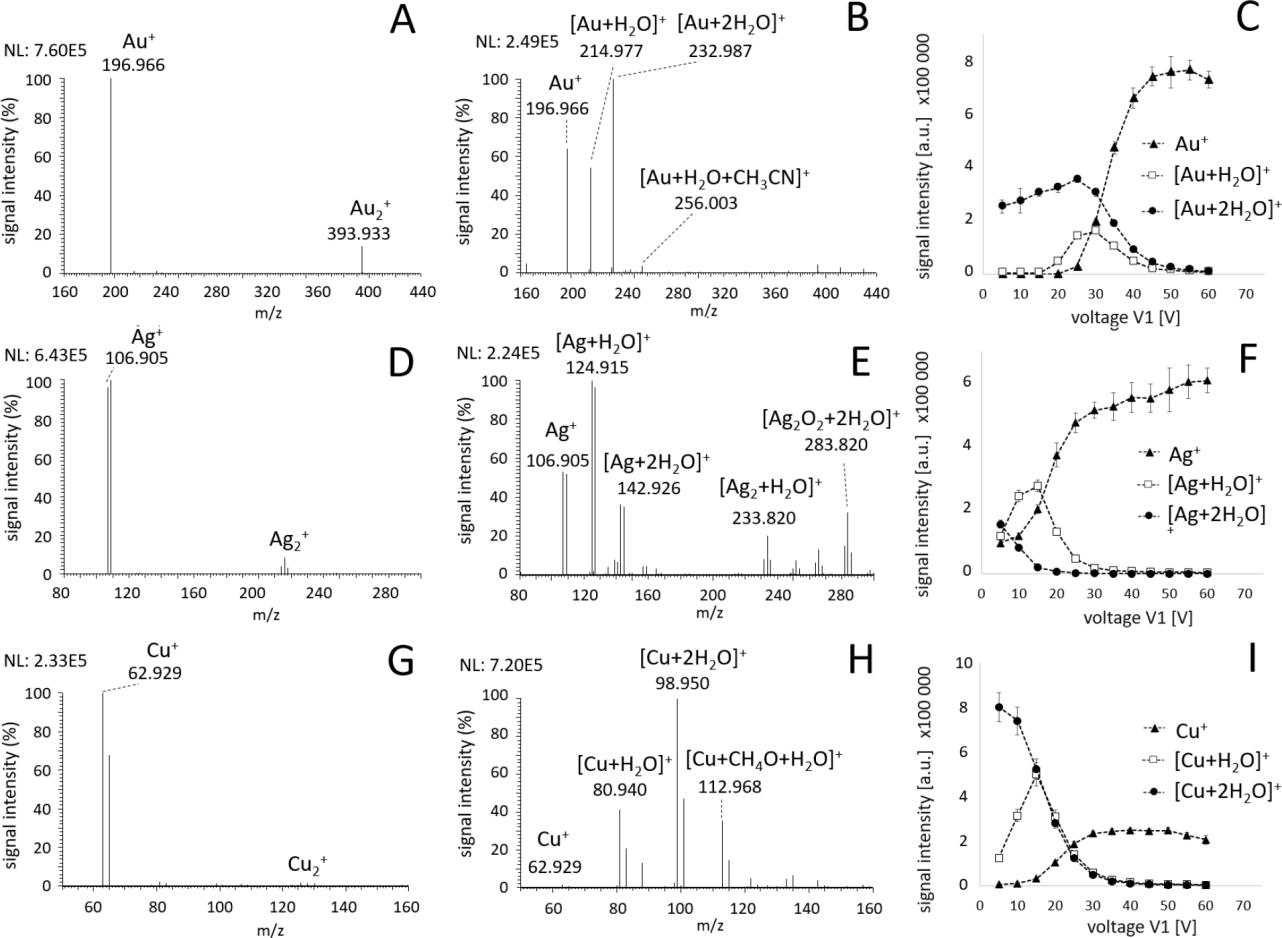
SubAP LDI mass spectra of (A, B) 6 nm gold, (D, E) 10 nm silver, and (G, H) 8 nm copper nanolayers with ambient air entering the ion source through ESI capillary. Voltage *V*_1_ = 60 V (A, D, G) promotes in-source fragmentation of metal ion complexes. Multiple complexes of M^+^ ions with H_2_O and acetonitrile were observed at *V*_1_ values of 30 (B) and 10 V (E, H). Abundances of Au^+^ (C), Ag^+^ (F), and Cu^+^ (I) ions and complexes formed with H_2_O plotted versus voltage *V*_1_.

Blank LDI mass spectra of all three metals were measured with laboratory air influx through the ESI capillary into the ion source (Figure 2). No additional VOCs were introduced. At these conditions, the formation of several ion species was observed; the dominant signals were metal ions M^+^ and metal ion complexes [M + H_2_O]^+^ and [M + 2H_2_O]^+^ with water present in the ambient air. Other minor signals were identified as metal clusters M_*n*_^+^ and adducts of metal ions with VOCs present in the laboratory air (mostly ion [M + H_2_O + C_2_H_3_N]^+^ containing acetonitrile originating from the mobile phases used in HPLC instruments in the same laboratory).

The crucial factor influencing the formation of the metal ion adducts in the ion source was the voltage *V*_1_ at the end of the second funnel. For all metals, spectra were measured while *V*_1_ was varied from 5 to 60 V (beyond these values, the signal intensity of observed ions dropped significantly due to reduced transport efficiency through the ion funnels). The value of *V*_1_ = 60 V resulted in clean spectra containing only metal ions M_*n*_^+^ (Figure 2A,D,G), while the value of 10 V provided intense signals of ion–molecular adducts for all metals (Figure 2B,E,H). The ESI capillary leads the gas vapor to the beginning of the first funnel right against the inlet of the rough vacuum pumping. Thus, we hypothesize that the reaction of the metal ions and VOCs occurs mainly in the first funnel before the VOCs are drained by the pump. In the second funnel, formed complexes can collide with the background gas and dissociate again, forming atomic metal ions M^+^. The higher the *V*_1_ value (meaning the lower the potential difference across the second funnel), the longer the time ions spend in the second funnel, and more collisions occur. The signal intensities of M^+^ ions and corresponding adducts with water plotted versus the *V*_1_ value are shown in Figure 2C,F,I. The maximum intensity of formed adducts [M + 2H_2_O]^+^ was observed at *V*_1_ values of 20, 5, and 10 V for Au, Ag, and Cu, respectively. This is in agreement with the published values of the bond energies of Au^+^, Ag^+^, and Cu^+^ ions with water molecules, which increase in the order Ag^+^, Cu^+^, Au^+^. The first H_2_O molecule bond energies with Ag^+^, Cu^+^, and Au^+^ ion are 134 ± 8, 161 ± 8, and 168 ± 10 kJ mol^–1^, respectively. The second H_2_O molecule bond energies for these adducts are 127 ± 8, 170 ± 7, and 188 ± 14 kJ mol^–1^, respectively.^30^

Gold, as a monoisotopic element, produces a dominant Au^+^ ion at *m*/*z* = 196.966 in the in-source fragmentation mode (*V*_1_ = 60 V, Figure 2A), while the single major species in the complex formation mode was the [Au + 2H_2_O]^+^ ion at *m*/*z* = 232.987. Figure 2B shows multiple adducts formed at *V*_1_ = 30 V. Similar behavior was observed also for LDI of Ag (Figure 2C,D) and Cu (Figure 2E,F) nanolayers. Both these elements have two naturally occurring isotopes with abundances of 51.8 (^107^Ag) and 48.2% (^109^Ag) and of 69.2 (^63^Cu) and 30.8% (^65^Cu), facilitating straightforward identification of formed metal ion adducts. The Ag^+^ ion formed predominantly a complex with a single water molecule [Ag + H_2_O]^+^ at *m*/*z* = 124.915, as its bond energy is significantly lower compared to Cu^+^ and Au^+^ complexes with water.

### Ionization of Selected VOCs

The following experiments focused on the ionization of selected VOCs, namely ethanol, acetone, acetic acid, cyclohexane, and xylene, using all three metals. For each compound, the resulting spectra are shown, the produced ions are labeled, and types of produced ions are summarized in Table 1. Possible reaction mechanisms leading to the formation of these ions are also proposed (see the Supporting Information).

**Table 1:** Species Identified in Spectra after Interaction of VOCs with Metal Ions M^+^^a^

M^+^	VOC	[M + VOC]^+^	[M + VOC + H_2_O]^+^	[M + 2VOC]^+^	[M + VOC – H_2_]^+^	[M + VOC – H_2_O]^+^	VOC^+^	fragments^b^
Au^+^	ethanol	–	++	++	++	++	–	–
	acetic acid	–	++	++	–	++	–	–
	acetone	–	++	++	–	–	–	–
	xylene	–	++	++	–	–	++	–
	cyclohexane	–	–	–	++	–	++	+
Ag^+^	ethanol	++	++	++	–	–	–	–
	acetic acid	++	++	++	–	–	–	–
	acetone	++	++	++	–	–	–	–
	xylene	++	++	++	–	–	–	–
	cyclohexane	–	–	–	–	–	–	–
Cu^+^	ethanol	–	++	++	+	+	–	–
	acetic acid	–	++	++	+	+	–	–
	acetone	–	++	++	–	–	–	–
	xylene	–	++	++	–	–	+	–
	cyclohexane	–	–	–	–	–	+	–

a ++, major ions; +, minor ions; −, ion not observed.

b Other reaction products involving breaking the C–C bond. This reaction was generally observed with Au^+^ ions and VOCs with longer aliphatic chains (data not shown).^17^

#### Ethanol

**Figure 3:**
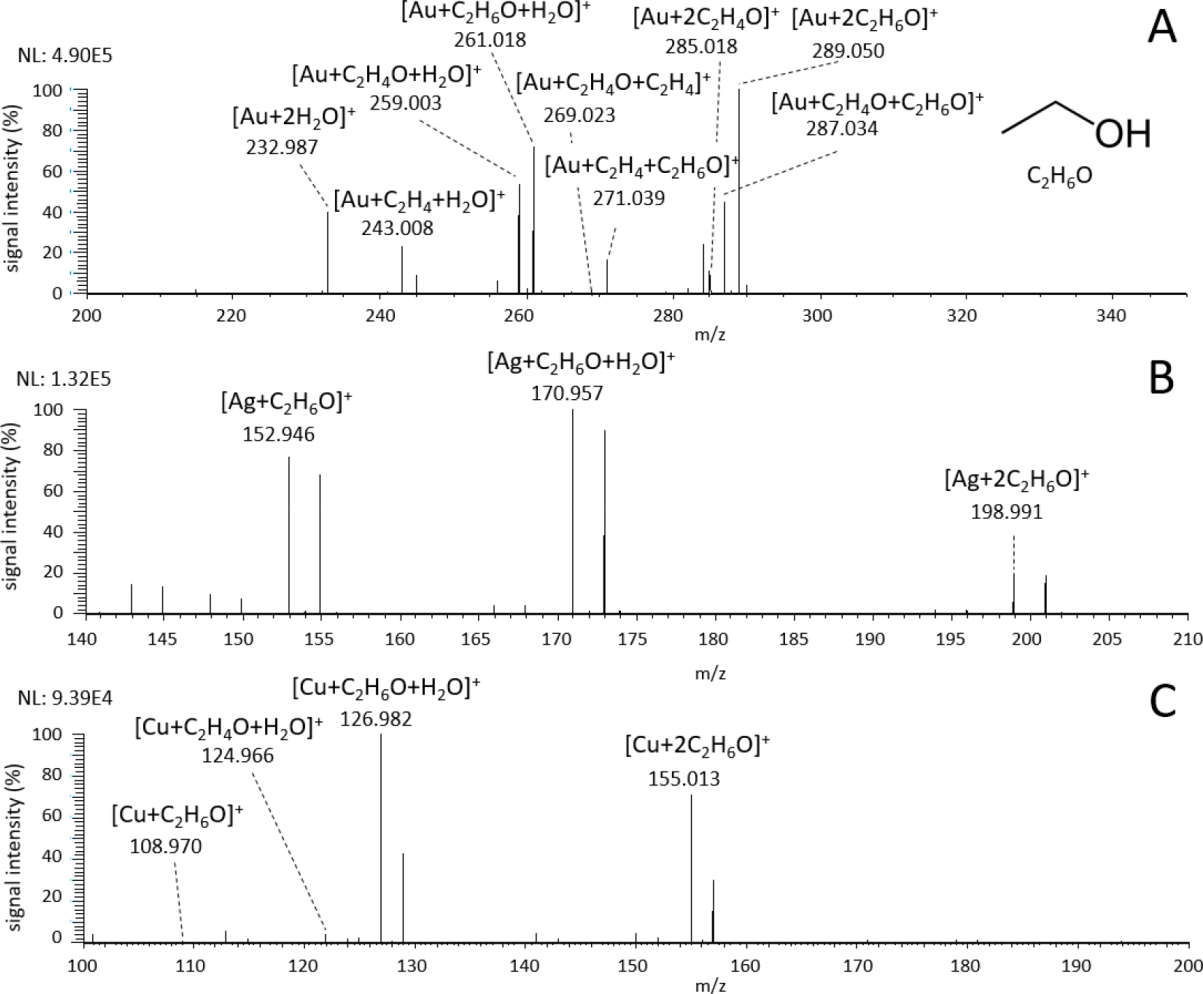
Mass spectra of ethanol vapors introduced into the ion source during LDI of (A) Au, (B) Ag, and (C) Cu nanolayers.

Ethanol was chosen as a representative of alcohols. Spectra obtained with ionization by Au^+^ ions are more complex compared to spectra with Ag^+^ and Cu^+^ ions; see Figure 3. This is due to the known ability of Au^+^ ion to activate C–C and C–H bonds^31,32^ resulting in the formation of multiple species after the dehydrogenation and dehydration of ethanol. These reactions were already discussed in our previous work.^19^ Briefly, the dominant species formed were [Au + C_2_H_6_O + H_2_O]^+^ and [Au + 2C_2_H_6_O]^+^. By dehydrogenation, the ions [Au + C_2_H_4_O + H_2_O]^+^, [Au + C_2_H_6_O + C_2_H_4_O]^+^, and [Au + 2C_2_H_4_O]^+^ were produced. Dehydration resulted in the formation of ions [Au + C_2_H_4_ + H_2_O]^+^, [Au + C_2_H_6_O + C_2_H_4_]^+^, and [Au + C_2_H_4_O + C_2_H_4_]^+^, where dehydrogenation also occurred. Though the [Au + C_2_H_4_ + H_2_O]^+^ complex is indistinguishable from the [Au + C_2_H_6_O]^+^ complex, which can be also formed by the loss of water bound in the [Au + C_2_H_6_O + H_2_O]^+^ complex, the presence of the [Au + C_2_H_4_]^+^ ion at *m*/*z* 224.997 (low intensity, not highlighted in the spectrum) indicates the reaction involving the dehydration of ethanol. The full list of the observed reactions is provided in the Supporting Information.

Ionization by Ag^+^ and Cu^+^ resulted in much simpler spectra, allowing straightforward identification of formed complexes. No reactions involving the loss of H_2_ or H_2_O were observed during the interaction of Ag^+^ and ethanol or higher alcohols (propanol, pentanol). The spectrum contained only three dominant complexes attributed to ethanol: [Ag + C_2_H_6_O]^+^, [Ag + C_2_H_6_O + H_2_O]^+^, and [Ag + 2C_2_H_6_O]^+^. In this case, the intense peak [Ag + C_2_H_6_O]^+^ can be attributed to a single ethanol molecule bound to Ag^+^ ion. The complexes [Ag + VOC]^+^ containing only one VOC ligand were also observed for other studied VOCs. Au and Cu complexes with one VOC ligand contained almost exclusively a molecule of water as well. Utilization of Cu^+^ ions resulted again in simpler spectra with two dominant species: [Cu + C_2_H_6_O + H_2_O]^+^ and [Cu + 2C_2_H_6_O]^+^. The dehydrogenation reaction resulting in the formation of ions [Cu + C_2_H_4_O + H_2_O]^+^ and [Cu + C_2_H_4_O]^+^ was observed, but to much less extent compared to usage of Au^+^ ions as reflected by the low intensities of these reaction products.

#### Acetic Acid

**Figure 4:**
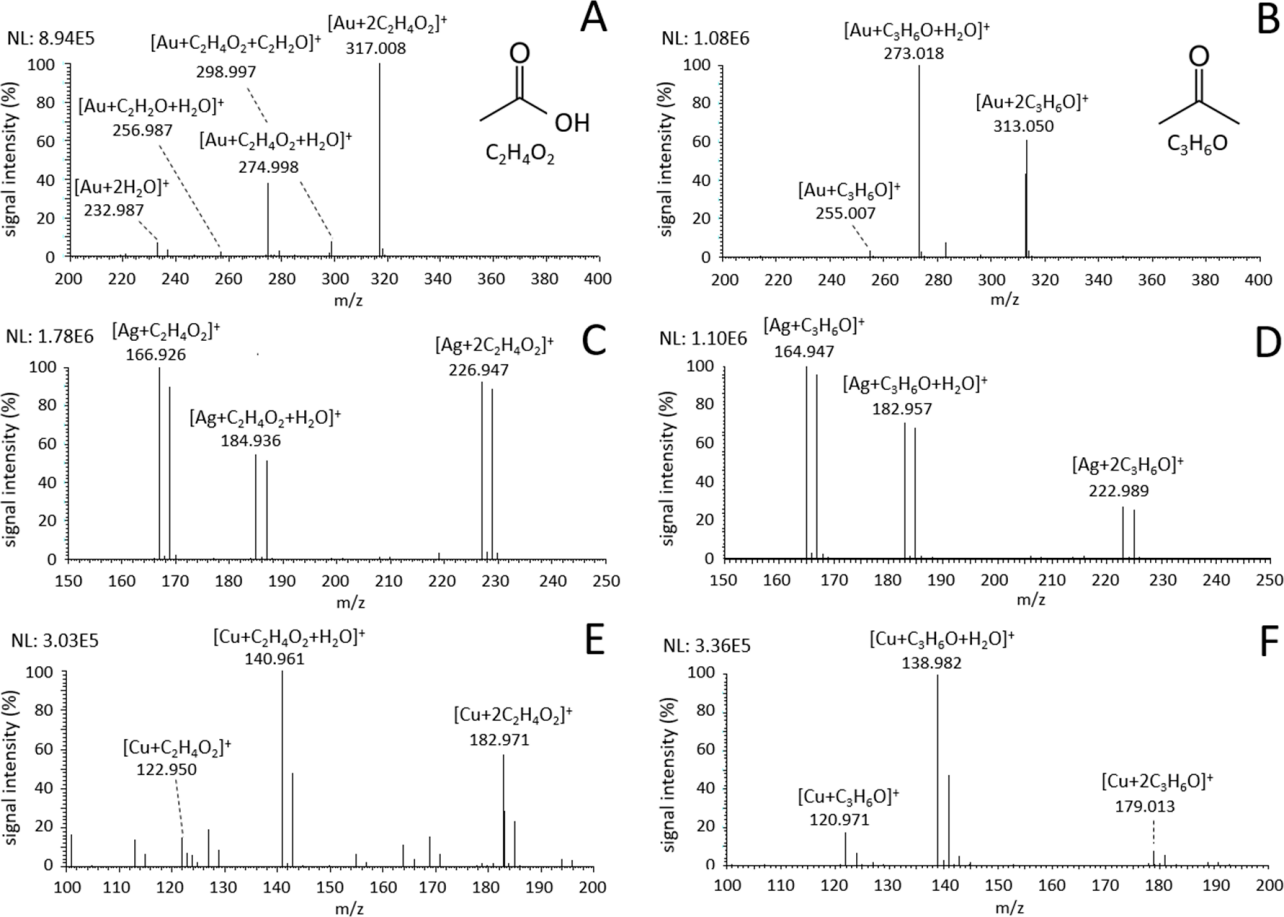
Mass spectra of (A, C, E) acetic acid and (B, D, F) acetone vapors introduced into the ion source during LDI of (A, B) Au, (C, D) Ag, and (E, F) Cu nanolayers.

Acetic acid was examined as a representative of carboxylic acids. Similarly to ethanol, the spectrum of acetic acid ionized by Au^+^ ions is more complex compared to ionization with Ag^+^ and Cu^+^ ions (Figure 4A,C,E). The prevalent reaction involved the loss of H_2_O from the molecule of acetic acid, which was most notable in the case of Au^+^ ion and very low in the cases of Ag^+^ and Cu^+^ ions. Loss of H_2_ is not possible from acetic acid, but this reaction was observed if carboxylic acids with longer carbon chains interacted with Au^+^ ion. Another reaction for carboxylic acids was the loss of CO. Products of this reaction were observed for all three metals, but only with marginal intensities. The spectra of all three metals are dominated by [M + C_2_H_4_O_2_ + H_2_O]^+^ and [M + 2C_2_H_4_O_2_]^+^; Ag^+^ formed, similarly to ethanol, also the intense ion complex [Ag + C_2_H_4_O_2_]^+^ with only one molecule of acetic acid bound without an additional molecule of water.

#### Acetone

Another studied group of VOCs included ketones, discussed in detail in the example of acetone (Figure 4B,D,E). Unlike ethanol and acetic acid, acetone produced only three types of ions with all three metals: [M + C_3_H_6_O]^+^, [M + C_3_H_6_O + H_2_O]^+^, and [M + 2C_3_H_6_O]^+^. The ion [M + C_3_H_6_O]^+^ was the dominant species in ionization by Ag^+^ and only a minor species in the cases of Au^+^ and Cu^+^ ions. It can be assumed that this species contains only a single molecule of acetone and no molecule of bound water, and it is not a product of the dehydrogenation reaction of acetone, as no other ions containing dehydrogenation products were observed. Higher ketones (pentanone and heptanone) provided additional products of dehydrogenation and, to a lesser extent, dehydration with Au^+^ ions. With Cu^+^ ions, only dehydrogenation occurred, and no reactions were observed with Ag^+^ ions, even for higher ketones.

#### Xylene

**Figure 5:**
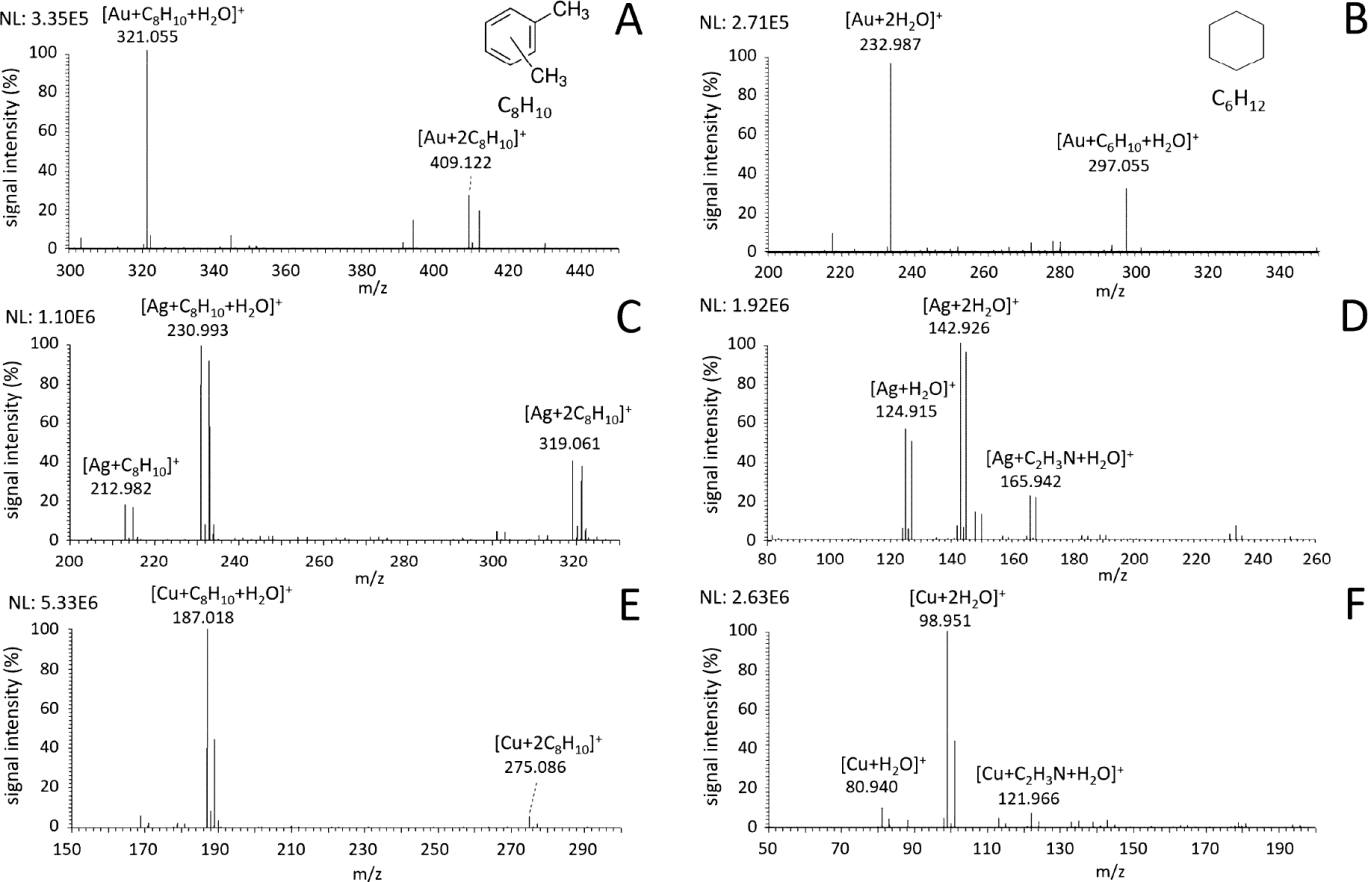
Mass spectra of (A, C, E) xylene and (B, D, F) cyclohexane vapors introduced into the ion source during LDI of (A, B) Au, (C, D) Ag, and (E, F) Cu nanolayers.

Aromatic compounds were represented by xylene. Ion–molecular complexes with compositions [M + C_8_H_10_ + H_2_O]^+^ and [M + 2C_8_H_10_]^+^ were observed for all three metals; see Figure 5. Additionally, Ag^+^ also formed the complex [Ag + C_8_H_10_]^+^, similar to other investigated VOCs discussed above. The spectra contained also ions without complexed metals including [C_8_H_7_]^+^, [C_8_H_8_]^+^, [C_8_H_9_]^+^, [C_8_H_10_]^+^, [C_8_H_11_]^+^, [C_7_H_7_]^+^, [C_7_H_9_]^+^, and [C_9_H_11_]^+^; see Figure S3A. These ions, with exact structures unknown, are products of several reactions: hydride abstraction, proton transfer, and presumably cleavage or addition of the −CH_3_ group.

#### Aliphatic Hydrocarbons

Aliphatic hydrocarbons present a group of VOCs which formed ion metal complexes only with Au^+^ ions. These compounds do not contain any binding groups (heteroatoms or multiple bonds) to interact with metal ions. Only Au^+^ ions can react through hydride abstraction, forming a double bond and subsequently complexes with the general formula [Au + 2C_*m*_H_*n*_ – H_2_ + H_2_O]^+^. Cyclohexane was introduced to the source, and dominant ions attributed to cyclohexane, [Au + C_6_H_10_ + H_2_O]^+^, were observed; see Figure 5B. A complex with two molecules of dehydrogenated cyclohexane, [Au + 2C_6_H_10_]^+^, was present with a very low signal at the given conditions. Similarly to aromatic compounds, ions without complexed Au^+^, [C_5_H_7_]^+^, [C_6_H_7_]^+^, [C_6_H_9_]^+^, [C_6_H_11_]^+^, [C_6_H_12_]^+^, and [C_7_H_13_]^+^, were also present; see Figure S3B. Cleavage of C–C bond was also occurring, but it is more important in the case of noncyclic hydrocarbons; see our previous work for more details.^19^ Utilization of Ag^+^ and Cu^+^ ions in this case did not produce any complexes with cyclohexane due to lesser reactivity (Figure 5D,F). Cu^+^ produced a weak signal (2–3 orders of magnitude lower compared to Au^+^ ions) of [C_6_H_9_]^+^, [C_6_H_11_]^+^, and [C_6_H_12_]^+^ ions. Interestingly, the addition of nonreacting aliphatic hydrocarbon vapor changed the relative intensities of Ag^+^ and Cu^+^ complexes with H_2_O. The intensity of ion [M + 2H_2_O]^+^ increased and and that of [M + H_2_O]^+^ decreased proportionally; see Figure S4.

### Influence of the Voltage *V*_1_ on the Formation of Ion–Molecular Complexes with Metals

One of the key parameters that affect the formation of the ion–molecular complexes with metals in the used subAP MALDI/ESI is the voltage *V*_1_ applied on the last electrode of the ion funnels. The voltage *V*_1_ value was changed from 5 to 60 V, and the spectra of four representatives of VOCs, ethanol, acetic acid, acetone, and xylene, were measured using Au^+^, Ag^+^, and Cu^+^ ionization. The graphs showing the intensities of the dominant formed species plotted against voltage *V*_1_ are provided in Figures S5–S7 for all VOCs.

**Figure 6:**
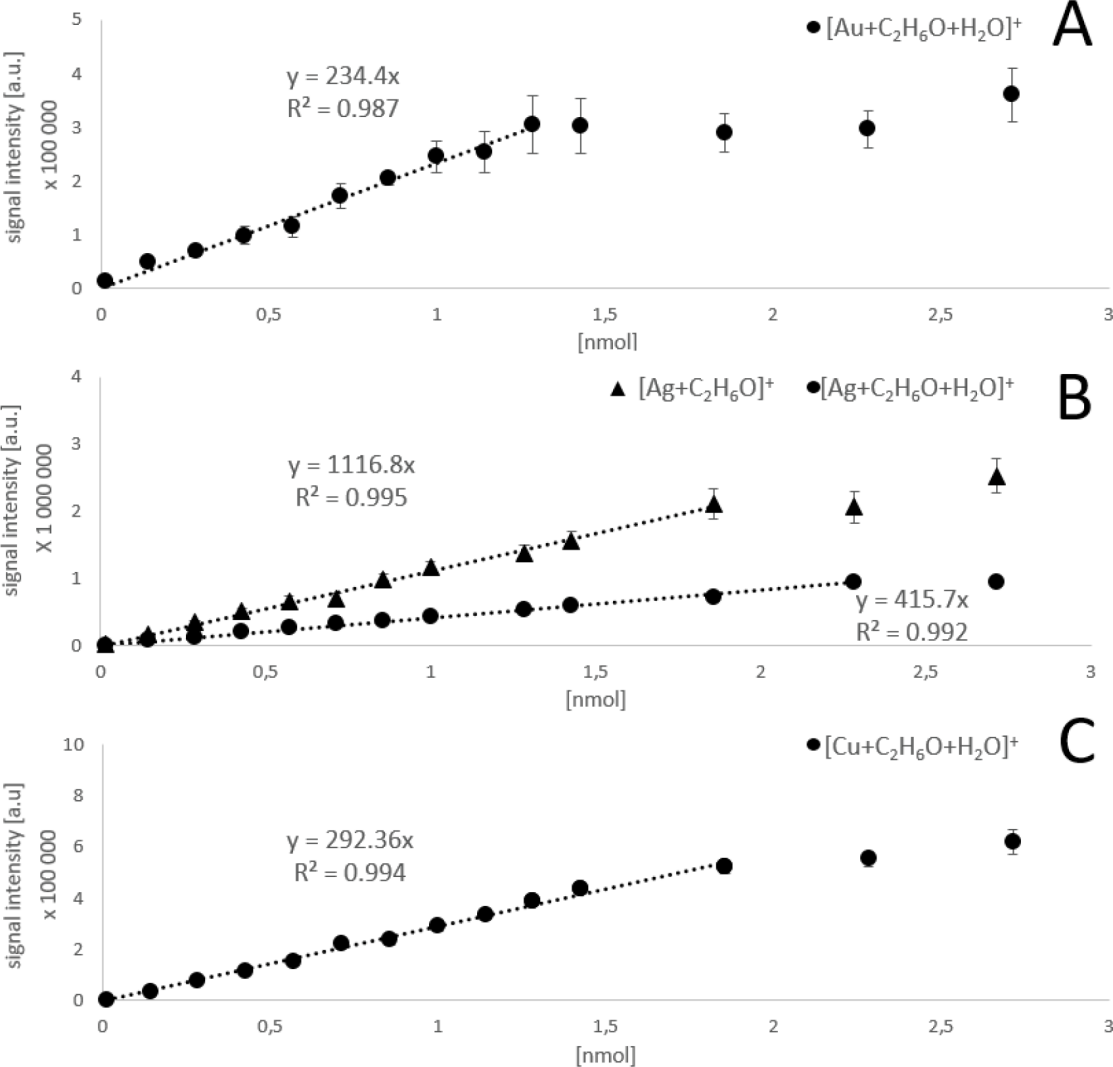
Calibration curves for ethanol with (A) Au^+^, (B) Ag^+^, and (C) Cu^+^ ions showing signals of complexes with a single molecule of ethanol plotted versus the amount of ethanol introduced to the ion source per single spectra acquisition.

The optimal values of *V*_1_, where the highest intensities of ion [M + VOC + H_2_O]^+^ (and additionally [M + VOC]^+^ in the case of Ag) were obtained, were used for the construction of calibration curves of VOCs with M^+^ ions. These species were chosen, as their intensity is proportional to introduced VOC until all M^+^ ions are consumed by the gas-phase reactions. The examples of linear calibration curves obtained for complexes of M^+^ and ethanol are shown in Figure 6. The calibration curves for acetone and acetic acid are shown in Figures S8 and S9. In these experiments, water solutions containing 1.3 μmol/L–3 mol/L VOCs were directly introduced to the ion source by a syringe equipped with a quartz capillary (i.d. 40 μm, o.d. 110 μm) inserted ∼2 mm into the heated ESI capillary. The amount of VOC introduced to the ion source during one spectrum acquisition (IT = 500 ms) ranged from ∼0.1 pmol to 0.25 μmol. The linear response of [M + VOC + H_2_O]^+^ ion was obtained using all three metals in the range from 1.5 pmol to ∼1.5 nmol of VOCs introduced. Additionally, [Ag + VOC]^+^ followed the same trend but exhibited higher intensity than [Ag + VOC + H_2_O]^+^ and thus was used for the LOD calculation. Using the obtained curves and signal of the blank at the *m*/*z* of the selected species during the influx of laboratory air, the LODs were estimated for the detection of VOCs with all three metal ions; see Table 2. The LODs are recalculated to the concentration in the introduced air (nanomoles per liter) based on the air influx of 0.5 L/s calculated in our previous work.^19^ It is seen that Ag^+^ ions provide up to an order of magnitude better LOD compared to Au^+^ ions for ethanol and acetic acid, where multiple side reactions with Au^+^ ions occur, mitigating the signal of [M + VOC + H_2_O]^+^. On the other hand, the LOD of the [M + H_2_O + VOC]^+^ complex with acetone, which does not react with any of the used metal cations, is similar for Au^+^ and Cu^+^ ions (0.1 nmol/L) and lower for Ag^+^ (0.5 nmol/L). This might be associated with the relativistic stabilization of the Au^+^ complex.^27^

**Table 2:** Limits of Detection (LODs) Calculated for Ethanol, Acetic Acid, and Acetone for Ionization by Au^+^, Ag^+^, and Cu^+^ Ions

	LOD (nmol/L)		
	ethanol	acetic acid	acetone
[Au + H_2_O + VOC]^+^	0.9	1.4	0.1
[Ag + H_2_O + VOC]^+^	0.9	0.2	0.5
[Ag + VOC]^+^	0.4	0.1	0.5
[Cu + H_2_O + VOC]^+^	0.7	0.3	0.1

The Supporting Information also includes calibration curves for mixtures of acetone and ethanol (Figures S10 and S11). In these experiments, the amount of introduced acetone (563 pmol) or ethanol (571 pmol) was held constant, and the amount of the second VOC (ethanol, acetone) was increased from 1.1 pmol to 2.6 nmol. Despite the competition between the two VOCs, the obtained LODs were comparable to those of pure substances.

### Analysis of VOC Mixtures

**Figure 7:**
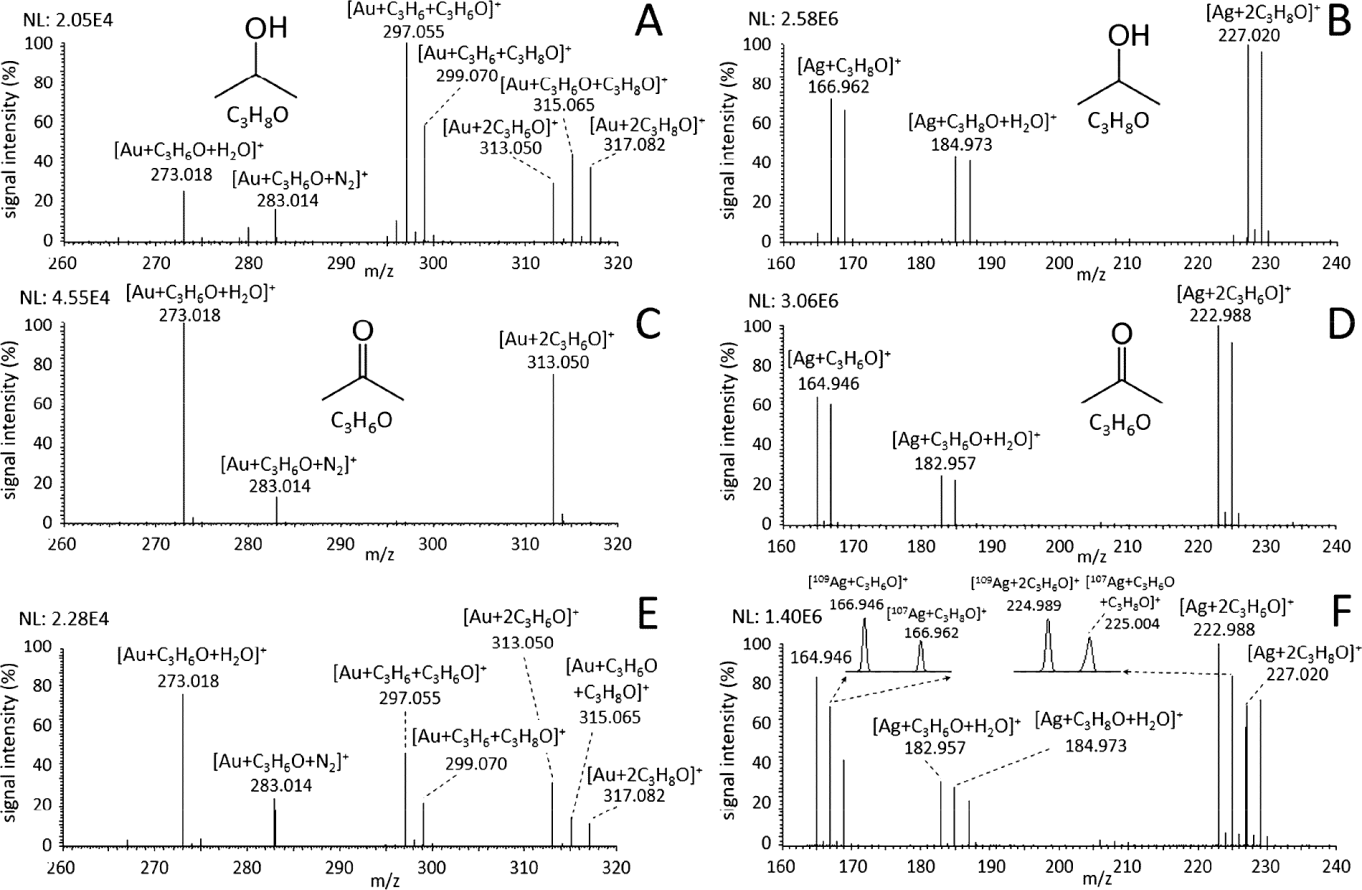
Mass spectra of (A, B) 2-propanol, (C, D) acetone, and their mixture (E, F) introduced into the ion source during LDI of (A, C, E) Au and (B, D, F) Ag nanolayers.

Introducing a mixture of VOCs to the ion source can present a significant challenge for the MIG technique, as the VOCs with different metal affinities compete for a limited number of charge bearing metal ions produced by LDI, especially when one VOC is present in large excess. In some cases, even detecting two VOCs in a mixture can be complicated for certain combinations of VOCs and metal ions. As an illustration, a mixture of 2-propanol and acetone was introduced to the ion source simultaneously by placing two beakers with the VOCs in the proximity of the inlet capillary. The acetone is indistinguishable in the mixture with propanol using Au^+^ ions because the ion–molecular complexes with the same summary formula are produced: 2-propanol forms characteristic [Au + C_3_H_8_O + H_2_O]^+^ and [Au + 2C_3_H_8_O]^+^ ions but also ions where hydride abstraction occurs, including [Au + C_3_H_6_O + H_2_O]^+^, [Au + C_3_H_6_O + C_3_H_8_O]^+^, and [Au + 2C_3_H_6_O]^+^. The latter ions overlap in the spectra with signature ions of acetone: [Au + C_3_H_6_O + H_2_O]^+^ and [Au + 2C_3_H_6_O]^+^ (see Figure 7A,C,E). On the other hand, utilization of nonreacting ions such as Ag^+^ produces characteristic signature ions for both compounds (Figure 7B,D). In the mixture, ions [Ag + C_3_H_8_O + H_2_O]^+^, [Ag + C_3_H_8_O]^+^, and [Ag + 2C_3_H_8_O]^+^ can be easily assigned to 2-propanol, while the ions [Ag + C_3_H_6_O + H_2_O]^+^, [Ag + C_3_H_6_O]^+^, and [Ag + 2C_3_H_6_O]^+^ are assigned to acetone (Figure 7F). The high resolving power of the orbital trap allows also distinguishing ions with isotopes ^107^Ag and ^109^Ag such as [^109^Ag + C_3_H_6_O]^+^ and [^107^Ag + C_3_H_8_O]^+^ containing acetone and 2-propanol, respectively. In this case, a similar pair of ions containing different ^107^Ag and ^109^Ag isotopes is seen at *m*/*z* 183, 225, and 227 (the two last also contain Ag^+^ ions with one molecule of 2-propanol and one molecule of acetone). Similar results were obtained also for a mixture of 1-butanol and 2-butanone (see Figure S12).

## Conclusion

We explored a utilization of Au^+^, Ag^+^, and Cu^+^ ions in a recently introduced MIG MS technique for the analysis of VOCs coupled to ultra-high-resolution mass spectrometry. Metal ions generated by LDI of a metal nanolayer give rise to ion–molecular complexes with molecules present in the surrounding gas at subAP conditions. These complexes are prone to fragmentation in the ion source. Thus, optimal conditions in the ion source must be selected to avoid fragmentation and, at the same time, efficiently transport to the MS instrument. In the case of the subAP MALDI/ESI source, this was achieved by a proper selection of voltages across the ion funnels.

The main difference between Au^+^ and the other two metal ions is its higher reactivity associated with the relativistic effects. Ag^+^ and Cu^+^ ions provided much simpler spectra and lower LODs compared to Au^+^ ions in the cases where Au^+^-induced side reactions occurred. Utilization of Ag^+^ and Cu^+^ ions allows identification of VOCs in mixtures, where Au^+^ ions react and produce substances with the same summary chemical as the other present VOC, for example, a pair of alcohol and ketone with the same number of C atoms in the molecule. Furthermore, both these elements have two stable isotopes, which can help in quick orientation in the spectra and identification of the metal-containing complexes in addition to the exact molecular formula provided by ultrahigh resolving power and sub-parts-per-million mass accuracy of the used mass spectrometer. On the other hand, the high Au^+^ ion reactivity is advantageous in detecting aliphatic hydrocarbons, which do not contain an electron-rich site where the M^+^ ion binds. Only usage of Au^+^ ions led to the detection of complexes containing dehydrogenation products of aliphatic hydrocarbons.

In general, the presented study encourages the utilization of metal ions of coinage elements for the ionization of small organic compounds. However, ionization is not the only possible application of the MIG technique. It can be expected that other transition metals, many of which possess catalytic properties, will also produce gas-phase complexes and induce gas-phase reactions similarly to the studied metals. Thus, the commercial dual subAP MALDI/ESI interface, used without any modifications, presents a general platform for studying the rich gas-phase chemistry of metal ions.

## Supporting Information Available

Details about the preparation of metal nanolayers and optimization of their thickness, additional mass spectra of xylene and cyclohexane ions formed without a complexed metal, spectra showing influence of nonreacting cyclohexane on Ag^+^ and Cu^+^ complexes with water, proposed reactions of studied VOCs with all metals, graphs showing the influence of voltages in the ion source on the formation of complexes with individual VOCs, calibration curves of acetic acid and acetone with all three metals, calibration curves of ethanol/acetone mixtures with Au^+^, comparison of 1-butanol/butanone mixture analysis with Au^+^ and Ag^+^ ions (PDF)
