# Supplementary material for: Comparison of Cu+, Ag+, and Au+ Ions as Ionization Agents of Volatile Organic Compounds at Subatmospheric Pressure
Source: J Am Soc Mass Spectrom. 2024 Jan 24;35(2):307–16. doi: 10.1021/jasms.3c00370 (PMC10853958; doi:10.1021/jasms.3c00370)
Supplement: Supplementary file 1 — js3c00370_si_001.pdf [file js3c00370_si_001.pdf]

Supporting Information

for

Comparison of Cu^+, Ag^+, and Au^+ ions as ionization agents of volatile organic compounds at sub-atmospheric pressure

Monika Koktavá, Vadym Prysiazhnyi, Jan Preisler, Antonín Bednařík *

Department of Chemistry, Faculty of Science, Masaryk University, Brno, Czech Republic

Corresponding author:

Antonín Bednařík

Department of Chemistry, Faculty of Science, Masaryk University

Kamenice 5, 625 00 Brno, Czech Republic

email: bednarik@mail.muni.cz

tel.: +420 549 49 6779

Table of Contents

1. Sputtering of metal nanolayers in the laboratory-built magnetron chamber S2
2. Optimization of metal layer thickness S2
3. Ions of xylene and cyclohexane without complexed metal S3
4. Influence of introduction of cyclohexane on LDI spectra with Ag^+ and Cu^+ S3
5. Proposed reactions of VOCs with metal ions S4
6. Influence of voltage V_1 on the formation of the metal adducts with volatile compounds in the ion source S7
7. Calibration curves S10
8. Calibration curves for mixtures of acetone and ethanol S12
9. Mixture of 1-butanol and butanone.............................. S15

1. Sputtering of metal nanolayers in the laboratory-built magnetron chamber

Ag and Cu films were prepared on microscopic slides using a laboratory-built magnetron sputtering device in a vacuum chamber provided by Activair, Czech Republic. The vacuum pumping system consisted of rotary and turbomolecular pumps, giving <10^{-4} Torr base pressure. The 3“ TORUS magnetron gun (Kurt J. Lesker, USA) with Ag or Cu target (99.99% pure, Camex s.r.o., Czech Republic) operated at 1.1 mTorr Pa pressure in Ar (99.996% pure) at 50 W or 150 W DC power, respectively, supplied by PD500X high-voltage power supply (Kurt J. Lesker, USA). The deposition rates were measured with the mechanical profilometer DektakXT using films deposited for 6, 12, and 18 min (Ag) and 3.5, 7, and 10.5 min (Cu), see Figure S1. A scratch was made on each film, and the height profile was measured three times. Based on the data, linear curves were obtained, and deposition rates of 10.5 and 4.6 nm/min were calculated for Ag and Cu, respectively.

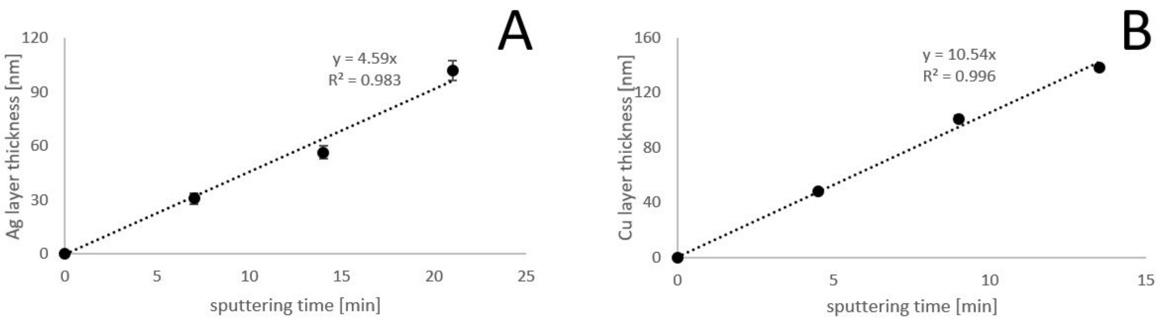

Figure S1: Deposition rate of A) Ag and B) Cu layer in the laboratory-built magnetron chamber.

2. Optimization of metal layer thickness

To select the optimal thickness of metal layers, i.e., the layers which yield the maximal amount of M^+ ions during LDI, several layers with calculated thicknesses of 1, 4, 6, 8, 10, and 20 nm (estimated by the deposition time and known deposition rate) were investigated. The average signal of M^+ was recorded in a 30 s linear laser scan over the substrate. Based on the data shown in Figure S2, the optimal layer thickness of 8, 10, and 6 nm was selected for Cu, Ag, and Au layers, respectively.

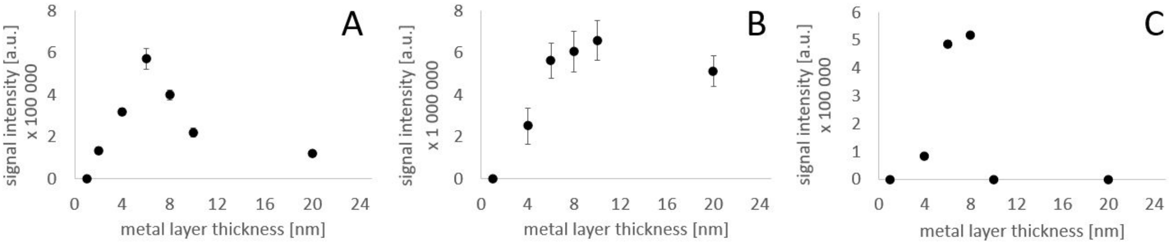

Figure S2: Intensity of A) Au^+, B) Ag^+, and C) Cu^+ ion-dependent on metal layer thickness.

3. Ions of xylene and cyclohexane without complexed metal

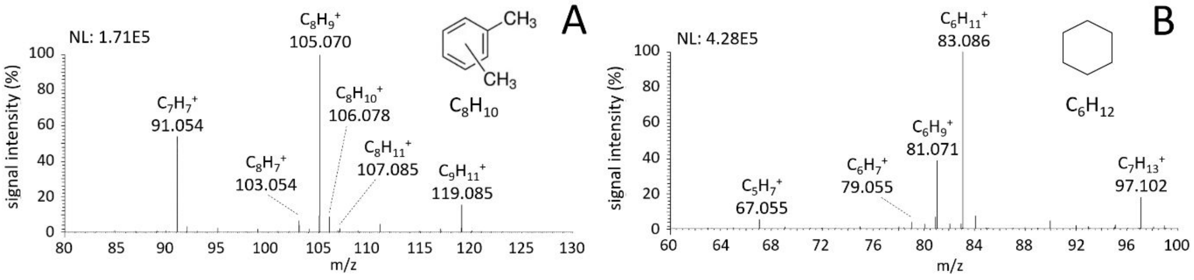

Figure S3: SubAP LDI mass spectra of A) xylene and B) cyclohexane ionized with Au^+.

4. Influence of introduction of cyclohexane on LDI spectra with Ag^+ and Cu^+

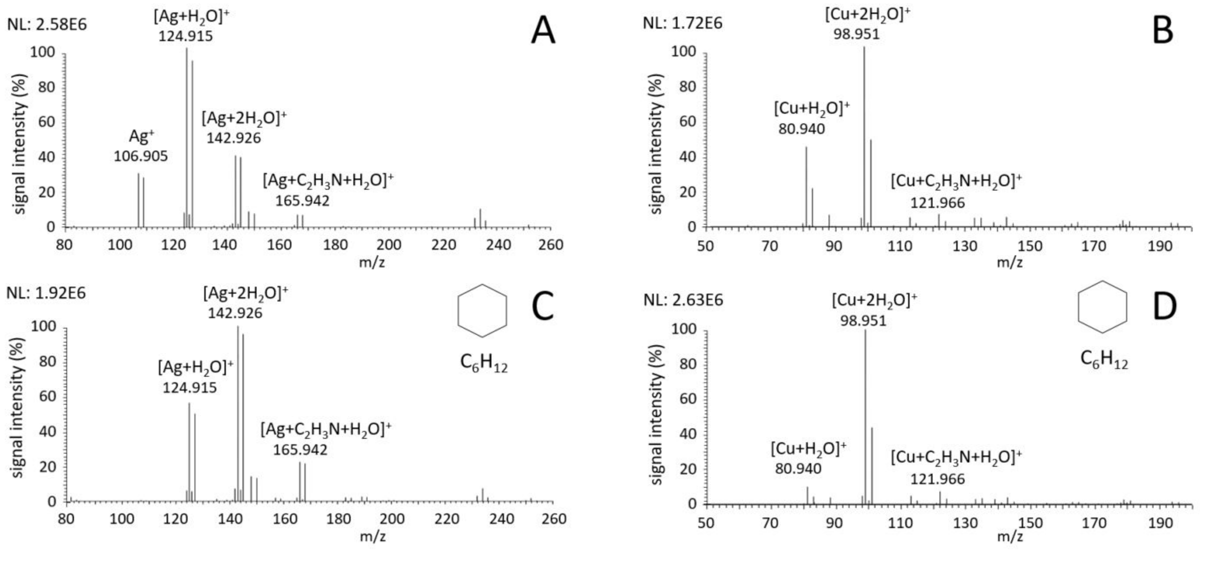

Figure S4: SubAP LDI mass spectra of A, C) 10 nm silver nanolayer and B, D) 8 nm copper nanolayer with ambient air A, B) and cyclohexane C, D) entering the ion source through ESI capillary.

5. Proposed reactions of VOCs with metal ions

Based on experiments with different values of voltage V1, signal intensities in MS and MS^2 spectra were proposed as possible reaction mechanisms for dominant signals.

Below is a list of reactions for selected VOCs with each metal, resulting in the formation of ion-molecular complexes, including the *m/z* of the observed ions (in bold). Spectra are shown in the figures 3-5 in the main text.

Ethanol:

$Au^+ + C_2H_6O + H_2O \rightarrow \mathbf{[Au + C_2H_6O + H_2O]^+}$	*m/z* 261.0181
$[Au + C_2H_6O + H_2O]^+ \rightarrow \mathbf{[Au + C_2H_4 + H_2O]^+} + H_2O$	*m/z* 243.0076
$[Au + C_2H_4 + H_2O]^+ \rightarrow \mathbf{[Au + C_2H_4]^+} + H_2O$	*m/z* 224.9972
$[Au + C_2H_6O + H_2O]^+ \rightarrow \mathbf{[Au + C_2H_4O + H_2O]^+} + H_2$	*m/z* 259.0026
$[Au + C_2H_6O + H_2O]^+ + C_2H_6O \rightarrow \mathbf{[Au + 2C_2H_6O]^+} + H_2O$	*m/z* 289.0495
$[Au + 2C_2H_6O]^+ \rightarrow \mathbf{[Au + C_2H_6O + C_2H_4O]^+} + H_2$	*m/z* 287.0339
$[Au + 2C_2H_6O]^+ \rightarrow \mathbf{[Au + C_2H_6O + C_2H_4]^+} + H_2O$	*m/z* 271.0389
$[Au + C_2H_6O + C_2H_4O]^+ \rightarrow \mathbf{[Au + C_2H_4O + C_2H_4]^+} + H_2O$	
$[Au + C_2H_6O + C_2H_4]^+ \rightarrow \mathbf{[Au + C_2H_4O + C_2H_4]^+} + H_2$	*m/z* 269.0235
$[Au + 2C_2H_6O]^+ \rightarrow \mathbf{[Au + 2C_2H_4O]^+} + 2H_2$	
$[Au + C_2H_6O + C_2H_4O]^+ \rightarrow \mathbf{[Au + 2C_2H_4O]^+} + H_2$	*m/z* 285.0183
$Ag^+ + C_2H_6O + H_2O \rightarrow \mathbf{[Ag + C_2H_6O]^+ + H_2O}$	*m/z* 152.9469
$Ag^+ + C_2H_6O + H_2O \rightarrow \mathbf{[Ag + C_2H_6O + H_2O]^+}$	*m/z* 170.9575
$[Ag + C_2H_6O + H_2O]^+ + C_2H_6O \rightarrow \mathbf{[Ag + 2C_2H_6O]^+} + H_2O$	*m/z* 198.9888
$Cu^+ + C_2H_6O + H_2O \rightarrow \mathbf{[Cu + C_2H_6O + H_2O]^+}$	*m/z* 126.9820
$[Cu + C_2H_6O + H_2O]^+ \rightarrow \mathbf{[Cu + C_2H_6O]^+} + H_2O$	*m/z* 108.9704
$[Cu + C_2H_6O + H_2O]^+ \rightarrow \mathbf{[Cu + C_2H_4O + H_2O]^+} + H_2$	*m/z* 124.9659
$[Cu + C_2H_4O + H_2O]^+ \rightarrow \mathbf{[Cu + C_2H_4O]^+} + H_2O$	*m/z* 106.9556
$[Cu + C_2H_6O + H_2O]^+ + C_2H_6O \rightarrow \mathbf{[Cu + 2C_2H_6O]^+} + H_2O$	*m/z* 155.0182

Acetic acid:

$Au^+ + C_2H_4O_2 + H_2O \rightarrow$ **[Au + $C_2H_4O_2$ + H_2O]$^+$** *m/z* 274.9977

[Au + $C_2H_4O_2$ + H_2O]$^+$ $\rightarrow$ **[Au + C_2H_2O + H_2O]$^+$** + H_2O *m/z* 256.9865

[Au + $C_2H_4O_2$ + H_2O]$^+$ + $C_2H_4O_2$ $\rightarrow$ **[Au + 2$C_2H_4O_2$]$^+$** + H_2O *m/z* 317.0082

[Au + 2$C_2H_4O_2$]$^+$ $\rightarrow$ **[Au + $C_2H_4O_2$ + C_2H_2O]$^+$** + H_2O *m/z* 298.9973

[Au + C_2H_2O + H_2O]$^+$ $\rightarrow$ **[Au + C_2H_2O]$^+$** + H_2O *m/z* 238.9763

[Au + $C_2H_4O_2$ + H_2O]$^+$ $\rightarrow$ **[Au + CH_6O_2]$^+$** + CO *m/z* 247.0026

$Ag^+ + C_2H_4O_2 + H_2O \rightarrow$ **[Ag + $C_2H_4O_2$ + H_2O]$^+$** *m/z* 184.9362

[Ag + $C_2H_4O_2$ + H_2O]$^+$ $\rightarrow$ **[Ag + $C_2H_4O_2$]$^+$** + H_2O *m/z* 166.9257

[Ag + $C_2H_4O_2$ + H_2O]$^+$ + $C_2H_4O_2$ $\rightarrow$ **[Ag + 2$C_2H_4O_2$]$^+$** + H_2O *m/z* 226.9468

[Ag + $C_2H_4O_2$ + H_2O]$^+$ $\rightarrow$ **[Ag + CH_6O_2]$^+$** + CO *m/z* 156.9412

$Cu^+ + C_2H_4O_2 + H_2O \rightarrow$ **[Cu + $C_2H_4O_2$ + H_2O]$^+$** *m/z* 140.9607

$Cu^+ + C_2H_4O_2 + H_2O \rightarrow$ **[Cu + $C_2H_4O_2$]$^+$** + H_2O *m/z* 122.9503

[Cu + $C_2H_4O_2$ + H_2O]$^+$ $\rightarrow$ **[Cu + C_2H_2O + H_2O]$^+$** - H_2O *m/z* 122.9503

[Cu + $C_2H_4O_2$ + H_2O]$^+$ + $C_2H_4O_2$ $\rightarrow$ **[Cu + 2$C_2H_4O_2$]$^+$** + H_2O *m/z* 182.9712

[Cu + $C_2H_4O_2$ + H_2O]$^+$ $\rightarrow$ **[Cu + CH_6O_2]$^+$** + CO *m/z* 112.9658

Acetone:

$Au^+ + C_3H_6O + H_2O \rightarrow$ **[Au + C_3H_6O + H_2O]$^+$** *m/z* 273.0182

[Au + C_3H_6O+ H_2O]$^+$ $\rightarrow$ **[Au + C_3H_6O]$^+$** + H_2O *m/z* 255.0070

[Au + C_3H_6O + H_2O]$^+$ + C_3H_6O $\rightarrow$ **[Au + 2C_3H_6O]$^+$** + H_2O *m/z* 314.0496

$Ag^+ + C_3H_6O + H_2O \rightarrow$ **[Ag + C_3H_6O + H_2O]$^+$** *m/z* 182.9571

[Ag + C_3H_6O+ H_2O]$^+$ $\rightarrow$ **[Ag + C_3H_6O]$^+$** + H_2O *m/z* 164.9465

[Ag + C_3H_6O + H_2O]$^+$ + C_3H_6O $\rightarrow$ **[Ag + 2C_3H_6O]$^+$** + H_2O *m/z* 222.9885

$Cu^+ + C_3H_6O + H_2O \rightarrow$ **[Cu + C_3H_6O + H_2O]$^+$** *m/z* 138.9815

[Cu + C_3H_6O+ H_2O]$^+$ $\rightarrow$ **[Cu + C_3H_6O]$^+$** + H_2O *m/z* 120.9706

[Cu + C_3H_6O + H_2O]$^+$ + C_3H_6O $\rightarrow$ **[Cu + 2C_3H_6O]$^+$** + H_2O *m/z* 179.0128

$Au^+ + C_2H_6O + H_2O \rightarrow$ **[Au + C_2H_6O + H_2O]$^+$** *m/z* 261.0181

Xylene:

$Au^+ + C_8H_{10} + H_2O \rightarrow$ **$[Au + C_8H_{10} + H_2O]^+$** *m/z* 321.0549

$[Au + C_8H_{10} + H_2O]^+ + C_8H_{10} \rightarrow$ **$[Au + 2C_8H_{10}]^+$** $+ H_2O$ *m/z* 409.1224

$Ag^+ + C_8H_{10} + H_2O \rightarrow$ **$[Ag + C_8H_{10} + H_2O]^+$** *m/z* 230.9931

$[Ag + C_8H_{10} + H_2O]^+ + C_8H_{10} \rightarrow$ **$[Ag + C_8H_{10}]^+$** $+ H_2O$ *m/z* 212.9824

$[Ag + C_8H_{10} + H_2O]^+ + C_8H_{10} \rightarrow$ **$[Ag + 2C_8H_{10}]^+$** $+ H_2O$ *m/z* 319.0606

$Cu^+ + C_8H_{10} + H_2O \rightarrow$ **$[Cu + C_8H_{10} + H_2O]^+$** *m/z* 187.0180

$[Cu + C_8H_{10} + H_2O]^+ + C_8H_{10} \rightarrow$ **$[Cu + 2C_8H_{10}]^+$** $+ H_2O$ *m/z* 275.0855

Cyclohexane:

$Au^+ + C_6H_{12} + H_2O \rightarrow$ **$[Au + C_6H_{10} + H_2O]^+$** $- H_2$ *m/z* 297.0549

$[Au + C_6H_{10} + H_2O]^+ + C_6H_{12} \rightarrow$ **$[Au + 2C_6H_{10}]^+$** $- H_2 + H_2O$ *m/z* 361.1225*

* Very low intensity of the product indicates a more complex multi-collision reaction pathway.

6. Influence of voltage V_1 on the formation of the metal adducts with volatile compounds in the ion source

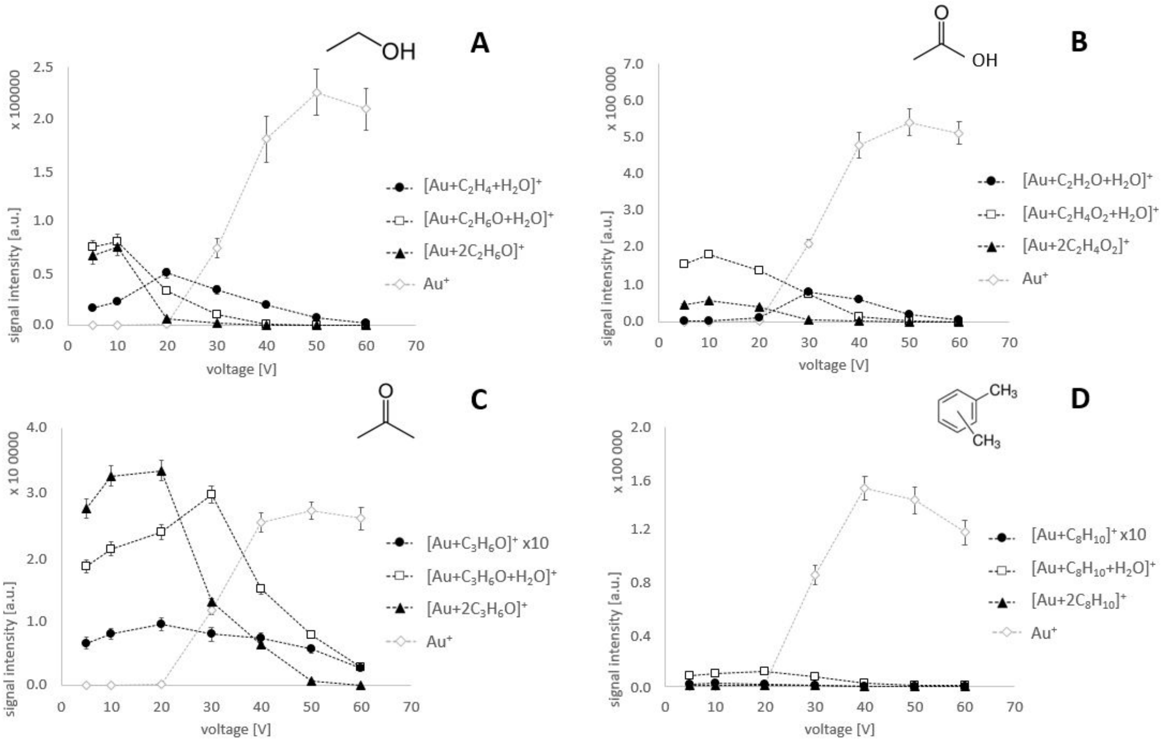

Figure S5: Dependence of intensities of ion-molecular complexes of A) ethanol, B) acetic acid, C) acetone, and D) xylene with Au on voltage V_1.

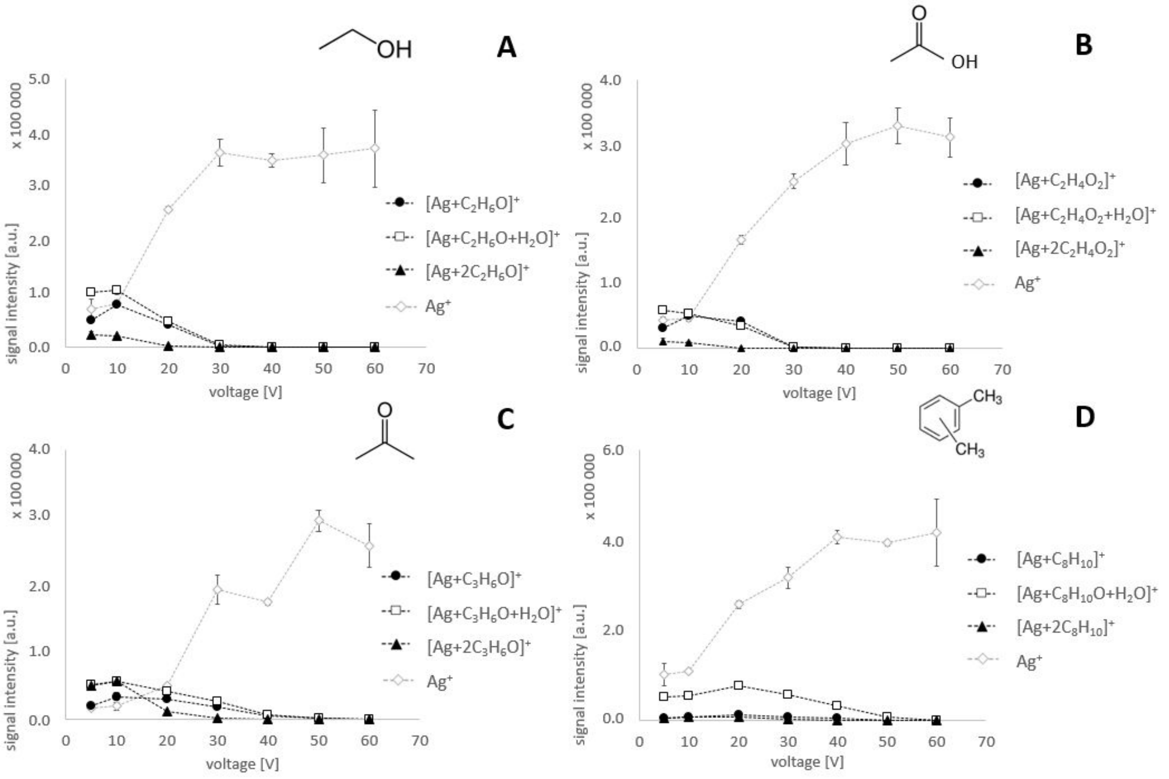

Figure S6: Dependence of intensities of ion-molecular complexes of A) ethanol, B) acetic acid, C) acetone, and D) xylene with Ag on voltage V_1.

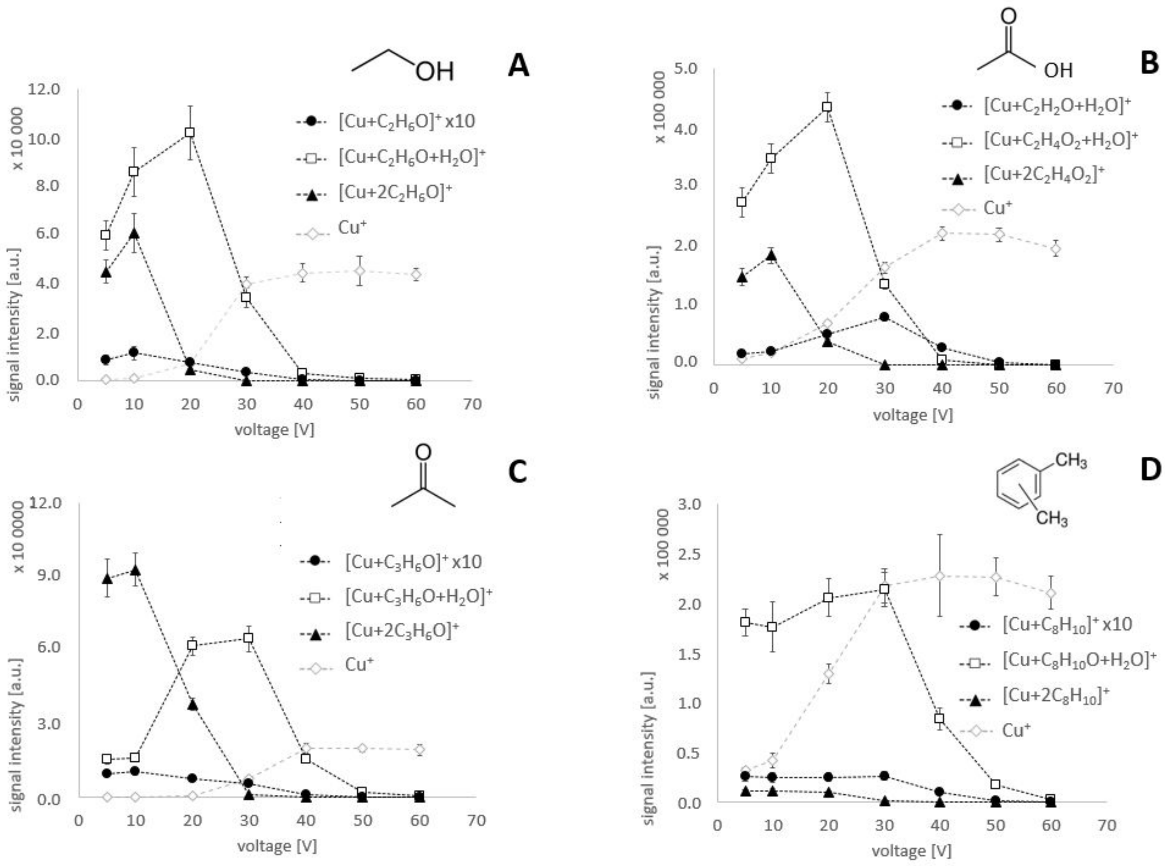

Figure S7: Dependence of intensities of ion-molecular complexes of A) ethanol, B) acetic acid, C) acetone, and D) xylene with Cu on voltage V_1.

7. Calibration curves

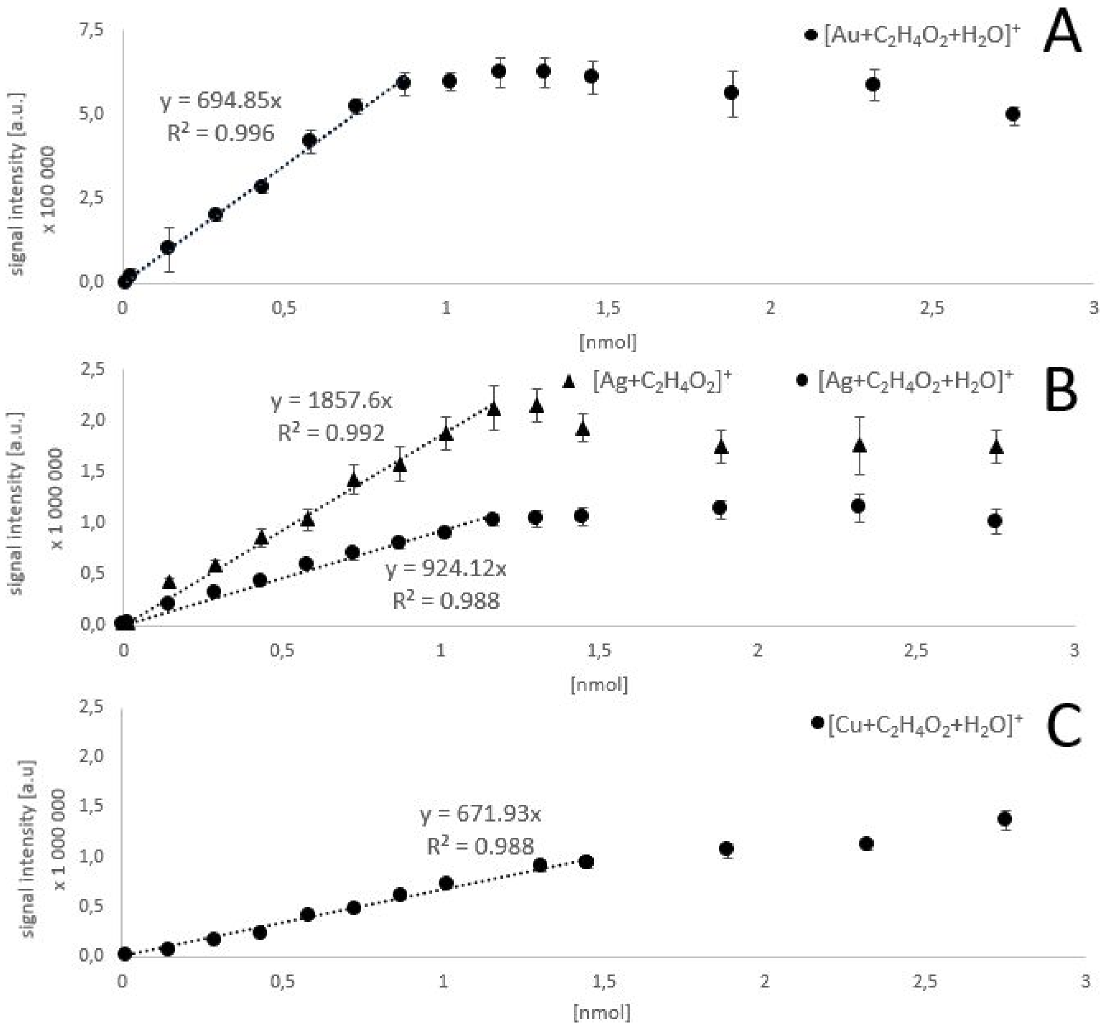

Figure S8: Calibration curves for acetic acid with A) Au^+, B) Ag^+, and C) Cu^+ ions.

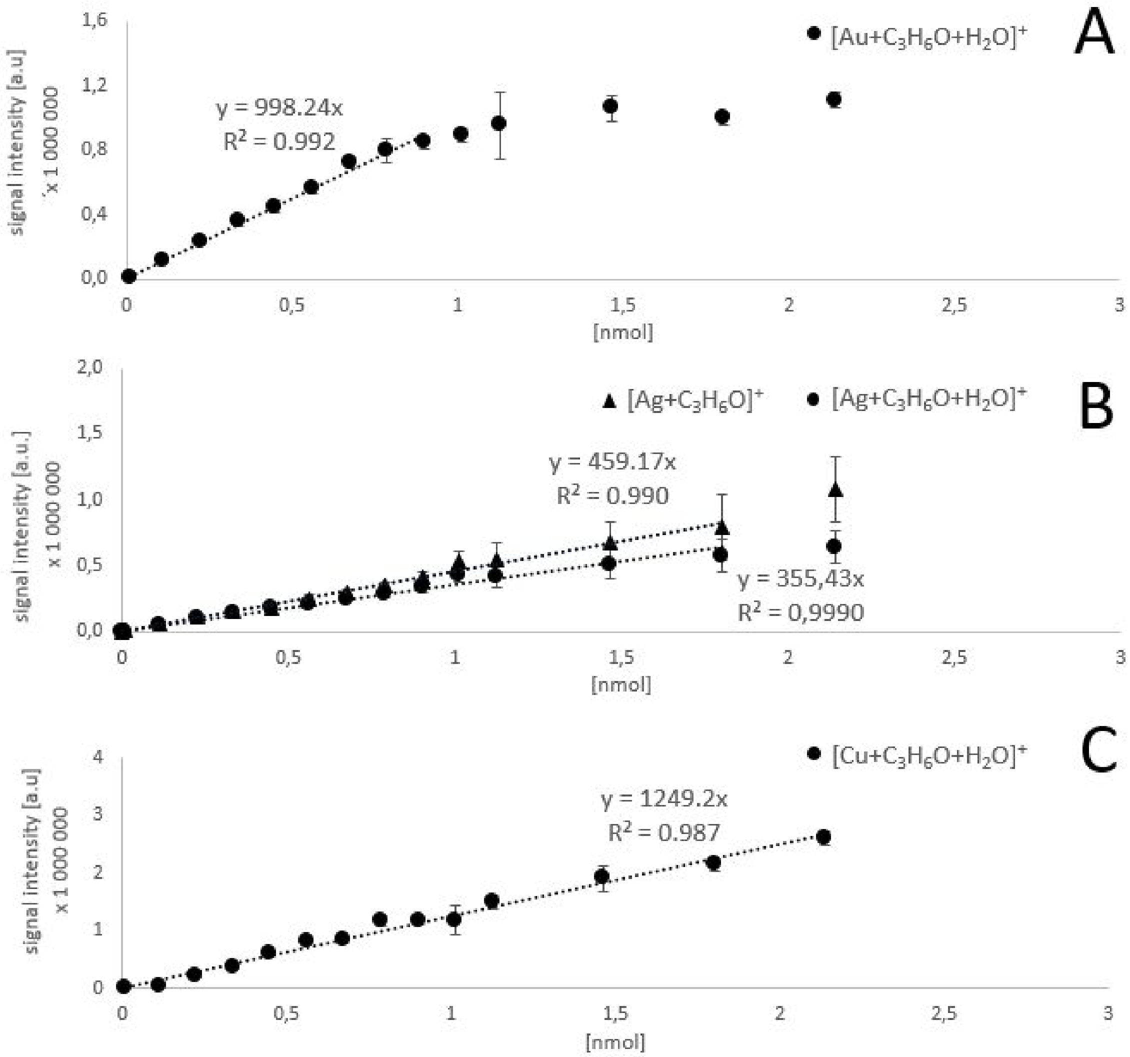

Figure S9: Calibration curves for acetone with A) Au^+, B) Ag^+, and C) Cu^+ ions.

8. Calibration curves for mixtures of acetone and ethanol

Determination of LODs was carried out with a mixture of two VOCs to address the effect of competition for M^+ ions. One VOC had a constant concentration, and the concentration of the other VOC was gradually increased. In the first case, solutions were introduced with a constant acetone concentration of 6.76 mmol/L and increasing ethanol concentrations of 17.1 µmol/L - 30.8 mmol/L. For the second experiment, the ethanol concentration was 6.85 mmol/L, and the acetone concentration increased between 13.5 µmol/L and 28.4 mmol/L. Figures S10 and S11 show data for ions containing only one type of VOCs, $[Au+C_2H_6O+H_2O]^+$, $[Au+C_3H_6O+H_2O]^+$, and the ion containing both VOCs, $[Au+C_3H_6O+C_2H_6O]^+$. The estimated LODs are shown in Table S1; these values are comparable to those for quantification from solutions of individual compounds. Of course, there is competition between VOCs for metal ions; if any VOCs were in excess, the quantitation of the others may not be possible. For example, acetone has a higher affinity for gold ions compared to ethanol, and the LODs for acetone are lower compared to ethanol. When increasing acetone concentration at a constant ethanol concentration (Figure S11), the signal intensity of acetone-containing ions grows rapidly, and the signal intensity of ethanol-containing ions drops sharply. In the opposite case, where ethanol concentration was increasing and acetone concentration was held constant, the intensity of acetone-containing ions decreased slowly (Figure S10).

Table S1: Limits of detection (LOD) calculated for ethanol or acetone in the mixture for ionization by Au^+.

	**LOD (nmol/L)**	
	**ethanol**	**acetone**
$[Au+H_2O+ C_2H_6O]^+$	1.7	
$[Au +H_2O+ C_3H_6O]^+$		0.2
$[Au+C_2H_6O+C_3H_6O]^+$	0.9	0.2

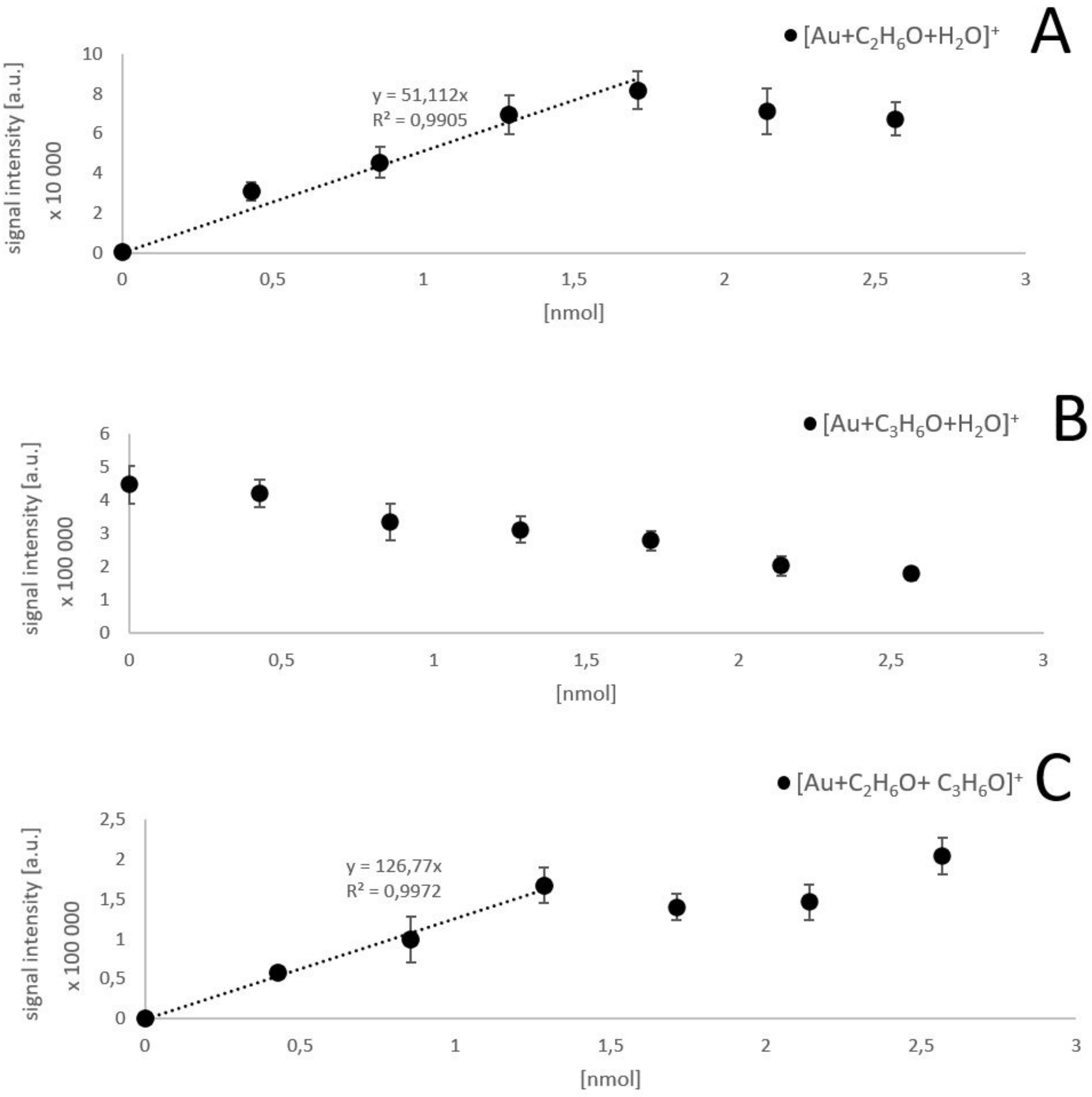

Figure S10: Calibration curves for mixture of acetone and ethanol showing dependence of A) $[Au+C_2H_6O+H_2O]^+$, B) $[Au+C_3H_6O+H_2O]^+$, and C) $[Au+C_2H_6O+H_2O]^+$ ion intensities on ethanol concentration. The concentration of acetone was kept constant; the amount corresponding to a single spectrum acquisition was 563 pmol. The amount of ethanol corresponding to a single spectrum acquisition ranged from 1.4 pmol to 2.6 nmol.

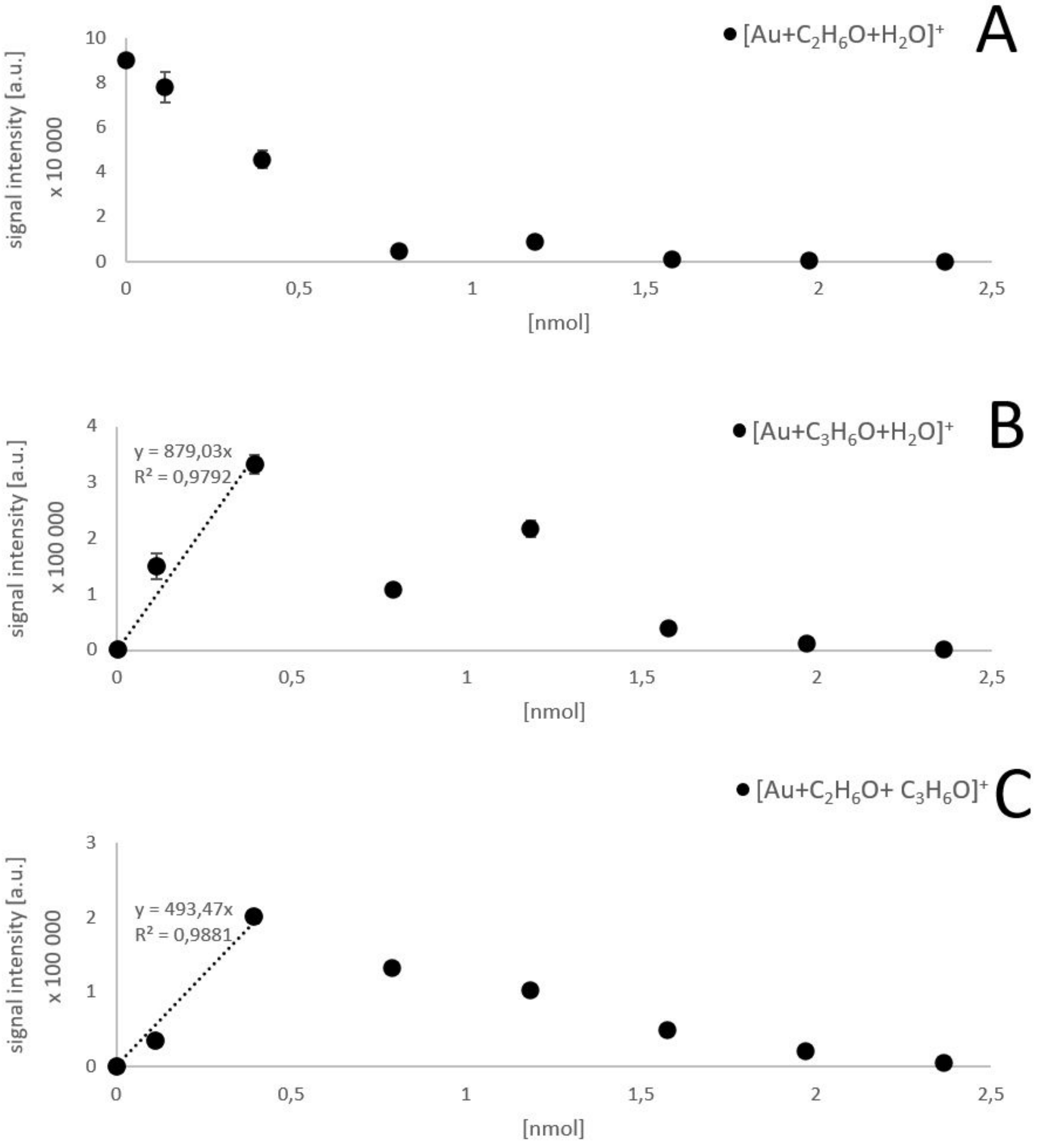

Figure S11: Calibration curves for mixture of acetone and ethanol showing dependence of A) [Au+C_2H_6O+H_2O]$^+$, B) [Au+C_3H_6O+H_2O]$^+$, and C) [Au+C_2H_6O+H_2O]$^+$ ion intensities on acetone concentration. The concentration of ethanol was kept constant; the amount corresponding to a single spectrum acquisition was 571 pmol. The amount of acetone corresponding to a single spectrum acquisition ranged from 1.13 pmol to 2.37 nmol.

9. Mixture of 1-butanol and butanone

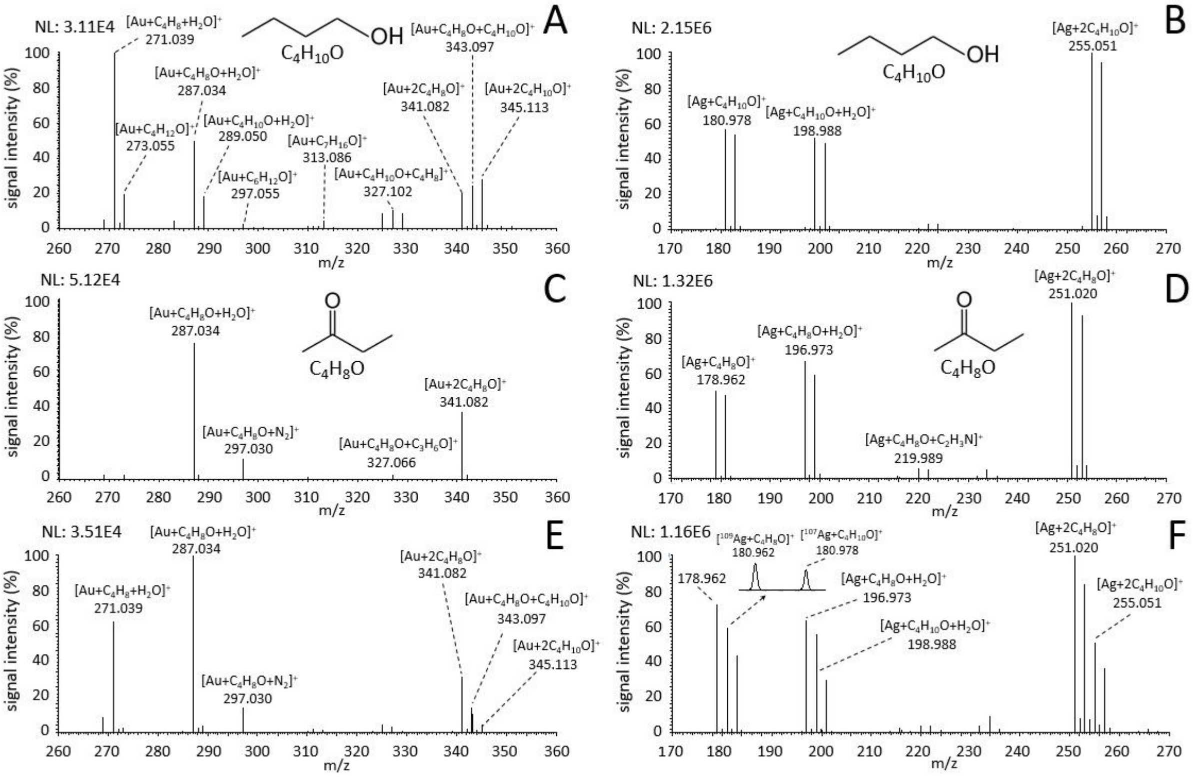

Figure S12: Mass spectra of A, B) 1-butanol, C, D) butanone and their mixture E, F) introduced into the ion source during LDI of A, C, E) Au, B, D, F) Ag nanolayer.
